# Perceptions of cervical cancer prevention among a group of ethnic minority women in Denmark—A qualitative study

**DOI:** 10.1371/journal.pone.0250816

**Published:** 2021-06-01

**Authors:** Sara Badre-Esfahani, Lone Kjeld Petersen, Camilla Rahr Tatari, Jan Blaakær, Berit Andersen, Lene Seibæk

**Affiliations:** 1 Department of Gynaecology and Obstetrics, Aarhus University Hospital, Aarhus N, Denmark; 2 Department of Public Health Programmes, Randers Regional Hospital, Randers NO, Denmark; 3 Department of Clinical Medicine, Aarhus University, Aarhus, Denmark; 4 Department of Gynaecology and Obstetrics, Odense University Hospital, Odense C, Denmark; 5 Open Patient data Explorative Network, University of Southern Denmark, Odense, Denmark; 6 Department of Clinical Research, University of Southern Denmark, Odense, Denmark; University of Wolverhampton, UNITED KINGDOM

## Abstract

**Background:**

Cervical cancer screening (CCS) and human papillomavirus vaccination (HPVV) are effective measures against cervical cancer (CC). Attendance in HPVV and CCS provides the greatest protection, while combined non-attendance in HPVV and CCS provides little to no protection. It is hence concerning that some large ethnic minority groups show considerably lower HPVV and CCS attendance than other women–especially women from Middle-Eastern and North African (MENA) countries and Pakistan. Little is, however, known about the reasons for this low combined attendance pattern n.

**Aim:**

To explore perceptions of and barriers to HPVV and CCS, among MENA and Pakistani women in Denmark.

**Method:**

Focus group interviews were conducted. Data was transcribed verbatim, and analysed using systematic text condensation.

**Findings:**

Seventeen long-term resident women originating from six major MENA countries and Pakistan were included. Mean age was 36 years. We found that these women, across different age groups and descent, had sparse knowledge and understanding about CC, and their perceived relevance of disease prevention was low. Compared to HPVV, their barriers to CCS were more fixed and often linked to socio-cultural factors such as taboos related to female genitals and sexuality. Moreover, they presented unmet expectations and signs of mistrust in the healthcare system. However, at the end of the interviews, participants became more attentive toward CC prevention, particularly toward HPVV.

**Conclusion:**

Elements of insufficient knowledge and understanding of CC and its prevention were found among a group of MENA and Pakistani women. Their socio-cultural background further represents a barrier particularly towards CCS. Additionally, negative experiences and unmet expectations lessen their trust in the healthcare system. All of which underlines the need for new tailored CC preventive strategies for this group. Based on our findings we suggest that future studies develop and evaluate interventions aiming to improve HPVV and CCS, including user-involvement.

## Introduction

Cervical cancer screening (CCS) has considerably reduced cervical cancer (CC) incidence and mortality [1, 2]. New vaccines targeting high risk human papillomavirus (HPV) have besides reduced HPV infections [3], as well HPV induced cell abnormalities [4].

Two universal and free-of charge services aimed at preventing HPV induced CC are on offer to residents of Denmark: a gender-neutral childhood HPV vaccination (HPVV) programme targeting all children aged 12–18 [5], and a CCS programme targeting all women aged 23–64 [6]. Both HPVV and CCS are typically provided by a general practitioner (GP) and the programmes have imbedded reminder services; however, citizens must actively book an appointment at their GP to receive the services.

To be best protected against CC, both HPVV and CCS are required. It is therefore concerning that HPVV and CCS attendance are substantially lower among some larger sub-populations, such as those with lower socio-economic status (SES) and ethnic minority background, particularly those from Middle Eastern and North African (MENA) countries or Pakistan [7–11].

Currently Denmark consists of 800,000 individuals with ethnic minority background, corresponding to 14% of the total Danish population; by 2060 this proportion is predicted to reach 21%. The largest and fastest growing minority group have migrated from non-Western countries, such as MENA and Pakistan, where the present number of immigrants (persons born outside Denmark) is 357,466 and descendants (persons born in Denmark with two immigrant parents) is 165,174 [12].

Ethnic minorities may face specific barriers, contributing to their relatively low degrees of HPVV and CCS attendance. European based qualitative studies, including one Danish study, investigating ethnic minority women’s barriers to CC prevention, have suggested that apart from practical, cognitive and lingual barriers, women from non-western countries may also face emotional or socio-cultural barriers to CCS and/or HPVV influencing their attendance [13–17]. These barriers include; stigma in relation to (cervical) cancer [13], women’s fear, embarrassment or shame in relation to CCS [15–17], barriers related to female genitals or sexuality [13, 15, 17], barriers related to religious beliefs/fatalism [13, 15] and low perceived risk [13, 16].

However, only few qualitative studies have investigating the interplay between HPVV- and CCS-specific perceptions and barriers among ethnic minority women in a post HPVV era [15, 17–19], and among these only two conducted in European settings [15, 17] and to date none have been conducted in Denmark.

The aim of this study was thus to explore perceptions of and potential barriers to HPVV and CCS among a group of ethnic minority women from MENA countries and Pakistan in Denmark.

## Methods and materials

### Design and participants

To address our aim, we applied a qualitative approach in accordance with the COREQ guidelines [20], that included focus group interviews (FGI) as data generation method. According to Kvale and Brinkmann [21] this technique makes it possible to gain insight into the participants’ understandings, experiences, and barriers. Furthermore, given that the FGI method stimulates dynamic conversations, we were able to observe variation and interplay between perceptions and barriers toward HPVV and CCS within the group [22].

The inclusion strategy was to include ethnic minority women from countries known to have the highest degree of combined HPVV and CCS non-attendance in Denmark, which in this case was MENA countries and Pakistan [11]. Furthermore, to include ethnic minority women who had both large insights into the Danish as well as their own ethnic society who were able to answer our research questions.

Inclusion criteria were thus women aged 23–65, with fluent Danish language skills and an ethnic minority background. Participants were either immigrants (born abroad) or descendants (born in Denmark), with parents originating from MENA and Pakistan, previously found to have the highest degree of combined non-attendance [11].

The sample size was in accordance with Malterud´s concept of Information Power [23], a method that strives to shift focus, in qualitative research, away from the quantity of participants (n) included towards the *quality* of the data provided by the participants. In studies with relative narrow aims, high quality communication between participants and interviewer, furthermore in studies which include participants with high level of knowledge and experience regarding the topic in question, relative few participants can be sufficient to secure high information power.

Eligible participants were recruited through snowball sampling [24] in Denmark’s second largest municipality, holding a population of approximately 350,000, including 16% with ethnic minority background. The participants were selected purposively to include as much variations in background characteristics as possible when recruiting participants known to be hard to reach [22].

The recruitment process involved assistance from key community informants, and prior local collaborators, such as “neighbourhood mothers” (a national volunteer/ambassador organization of ethnic minority women trained to help less advantaged women their communities addressing sensitive issues such as domestic violence, women’s rights, social control etc.). As well as bilingual healthcare professionals in the field of gynaecology. Furthermore, we posted advertisements to attend the project in country-specific Facebook groups (e.g. “Moroccans in Denmark”).

Potential participants were approached by Sara Badre-Esfahani (SBE) face-to-face or via telephone, and introduced to the aim of the study, and the framework of FGI´s. Once verbal agreement to participate had been given, all the participants received a short-written presentation of the study prior to participation.

### Interviews

Four Danish spoken FGI´s were conducted by SBE and supervised by Lene Seibæk (LS) between August and December 2019. A semi-structured interview guide was developed based on a priori knowledge on the field and used to guide the interviews (Table 1).

**Table 1:** The semi-structured interview guide.

*Topic*	*Subject Questions*
*Basic Knowledge*	What do you know about cervical cancer? How / why does one get the disease? Can one prevent the disease? How can one prevent the disease?
*Time-out 1*	Presentation of research results showing ethnic inequality in cervical cancer prevention
*Response to results*	Why do you think immigrant women from MENA countries and Pakistan participate less than women with Danish / other immigrant backgrounds? Do you think ethnic minority women should participate more often than they do now?
*Information sources*	Where do you get your knowledge of health and illness from? How would you prefer to have this knowledge (in Danish / in native language, in paper form / electronically)?
*Time-out 2*	Presentation about HPV, cervical cancer and screening and HPV vaccination
*Response to the presentation*	Was there anything in the presentation that you didn’t know before? Anything that surprised you? Do you think this new knowledge will affect your perception of cervical cancer prevention? Do you think this knowledge will change your participation in cervical cancer prevention?

Before the interviews, each participant filled in a background questionnaire that included information on socio-economics, demographics, and HPVV and CCS status of themselves and children, if relevant, combined with a form of consent (S1 and S2 Files).

Participants were then introduced to the FGI setting, and encouraged to share all aspects of their thoughts and perceptions, and to discuss their opinions and disagreements with each other. Initially, to frame and focus the FGI´s, the participants were presented with simple diagrams, based on previous results [11], illustrating the ethnic inequity in HPVV and CCS attendance in Denmark–particularly among women from MENA and Pakistan compared to native Danish and minority women from other regions of the world (S1 Fig). This presentation we called “Time-out 1” (Table 1).

Then they were asked to share their knowledge and understandings of CC and its prevention. Approximately halfway through each interview, a second time-out was introduced, where the participants were given a short presentation, based on simple on set hand drawings, illustrating the natural history of HPV-derived CC and the biomedical logics of HPVV and CCS. The purpose of this time-out was to build a “common ground” of knowledge concerning HPV and CC for further discussions.

### Analysis

The FGI´s were audio recoded with permission from all participants, on one condition that only the first and last author had access to the data. After each FGI these audio recordings were transcribed verbatim by SBE. All transcripts were then read systematically by SBE and LS, while listening to the audio recordings, to check the quality of the transcripts and gain an “overall impression of the data”.

The data were then analysed according the Malterud’s systematic text condensation (STC) [25] which is a pragmatic and stepwise thematic analysis developed especially for novice qualitative researchers, that guides the researcher from the transcript to the final presentation of findings. It consists of four main steps;

1. generating an overall impression of the data and defining preliminary findings,
2. de-contextualizing the transcript into small elements called; meaning units, and coding these in groups
3. re-contextualization these elements to a multi-vocal first-person narrative and, finally,
4. developing an analytic text and reaching a novel understanding of the research topic.

The initial steps of the STC analyses were conducted after the first two interviews, by SBE and LS, and the attention of the final two interviews was focused according to these preliminary findings. After the last interview, in-depth STC analyses were performed for all transcripts and findings discussed thoroughly by SBE, LS and co-author Camilla Rahr Tatari (CRT). As CRT had not participated in the FGI´s, nor had access to the raw data; her involvement at this point ensured transparency of the analysis process and validation of the findings. Finally, these findings were critically discussed among the entire author group.

### Ethics

Due to the sensitive nature of the topic, and the risk of revealing the identities of the participants, we fully anonymized the identities of the participants using solely identification numbers (P1-P17).

Because the FGI´s were expected to alter the participants’ perceptions of CC prevention, potentially creating new awareness and perhaps worries, the participants were offered a debriefing by telephone at the end of the study period by SBE. At these phone conversations SBE initiated by personally thanking the participants for their contributions, subsequently SBE asked each participant whether any questions, concerns or worries had a raised during or after the FGI, they need to discuss with SBE.

All participants filled out a written informed consent stating that the participants had received both verbal and written information about the study, and that background information and participant statements may be presented anonymously; it also specified that participants at any time had the right to withdraw their consent (S1 and S2 Files).

The study protocol was reported according to the EU’s General Data Protection Regulation (article 30), and listed in the record of processing activities for research projects in Central Denmark Region (J. No.: 1-16-02-400-16). The Regional Committee for Medical and Health Research Ethics were also contacted in line with local legislation (S3 File) and found that the study did not require formal ethical approval (J. No.: Date of decision 28-02-2017) (S4 File). The study furthermore followed the principles of The World Medical Associations’ declaration of Helsinki–Ethical principles for medical research involving human subjects.

## Results

Four FGI were conducted, participants representing six different MENA countries and Pakistan were included. Each FGI consisted of three to six participants, at locations near the participants’ homes or workplaces. The mean interview length was 83 minutes (Table 2).

**Table 2:** Characteristics of the focus group interviews.

Interview	Length	Location	Country of origin	No. of participants
FGI 1	85 minutes	Community house	Somalia	4
			Turkey	1
			Lebanon/Palestine	1
FGI 2	105 minutes	Community house	Somalia	1
			Turkey	2
			Lebanon/Palestine	1
FGI 3	65 minutes	Private home	Pakistan	4
FGI 4	77 minutes	Private Midwife centre	Iraq	1
			Morocco	1
			Tunisia	1
				*Total* 17

Table 3 illustrates self-reported background socio-economic characteristics besides CCS and HPVV status (of the participants and their children when relevant). The age ranged between 16 and 58 years, with a mean age of 36. Nine women were born in a MENA country, one was born I Pakistan, and seven born in Denmark. Thirteen out of fifteen women stated to have lived more than twenty years in Denmark. Six women were married, one widow, five divorced while four were single. Ten out of the seventeen participants had children; seven had up to three children, while three had up to seven children. Seven out of thirteen women stated to have a short educational level while four stated to have middle and two high educational level. Furthermore, eighth out of fifteen stated to be working, three not to be working while four stated to be studying at the time of the interviews.

**Table 3:** Self-reported background characteristics of the FGI participants.

**Age in years**		
< 23		2
23–40		7
> 40		6
*Missing*		*2*
**Civil status**		
Married		6
Divorced		5
Widowed		1
Single		4
*Missing*		*1*
**Country of origin**		
Somalia		5
Pakistan		4
Turkey		3
Palestine		2
Iraq		1
Tunisia		1
Morocco		1
**Migrations status**		
Immigrant		10
Descendant		7
**Number of children**		
0		7
1–3		7
4–7		3
**Years lived in Denmark**		
< 20		4
20–29		7
> 30		3
*Missing*		*3*
**Occupational status**		
Working		8
Not working		3
Student		4
*Missing*		*2*
**Educational level**		
Short		7
Middle		4
High		2
*Missing*		*4*
Yes		8
No		5
Not eligible		2
*Missing*		*2*
**Participants HPV-vaccination**		
Yes		3
No		0
Not eligible		12
*Missing*		*2*
**Participants children’s HPV-vaccination**		
Yes		6
No		1
Not eligible		10

Six out of seven participants with a child eligible for HPVV had agreed to vaccinate their children. Three out of fifteen participants had been eligible for HPVV themselves and all of these had initiated HPVV. Twelve had been eligible for CCS, of these eight had participated in minimum one CCS, four had not.

### Overall impression of the data

The atmosphere of the FGI´s were dynamic, positive and respectful. The participants came across as curious, focused and active. During the FGI´s they were able to present own perceptions and barriers openly, while discussing, absorbing and respecting each other’s various perceptions and barriers.

These characteristics gave rise to many rich and nuanced discussions. For example, one participant told about her prior negative experiences with breast cancer screening, while two other participants contributed by sharing their own positive experiences and addressing issues such as false positive and negative screening results.

One participant´s perceptions were fundamentally different from the others, as she presented high level of knowledge, no barriers or mistrust. While one participant remained passive and contributed substantially less than the other sixteen. However, in most cases, participants across all nationalities and age groups shared similar views, resulting in relatively few yet strong perceptions.

The perceptions and barriers initially presented were; insufficient knowledge regarding CC, lack of nuanced understating of HPVV/CCS, and low perceived risk and relevance of cancer prevention especially CCS. Besides socio-cultural barriers related to issue of the female genitals. However, towards the end of the interviews, as their knowledge and perceived relevance of HPVV and CCS improved, most participants expressed a more positive attitude towards cancer prevention especially HPVV. Furthermore, advocating a need for more targeted information and CC preventive efforts in their communities. Some even suggested implementing compulsory attendance to secure better compliance.

### Themes

**Fig 1:**
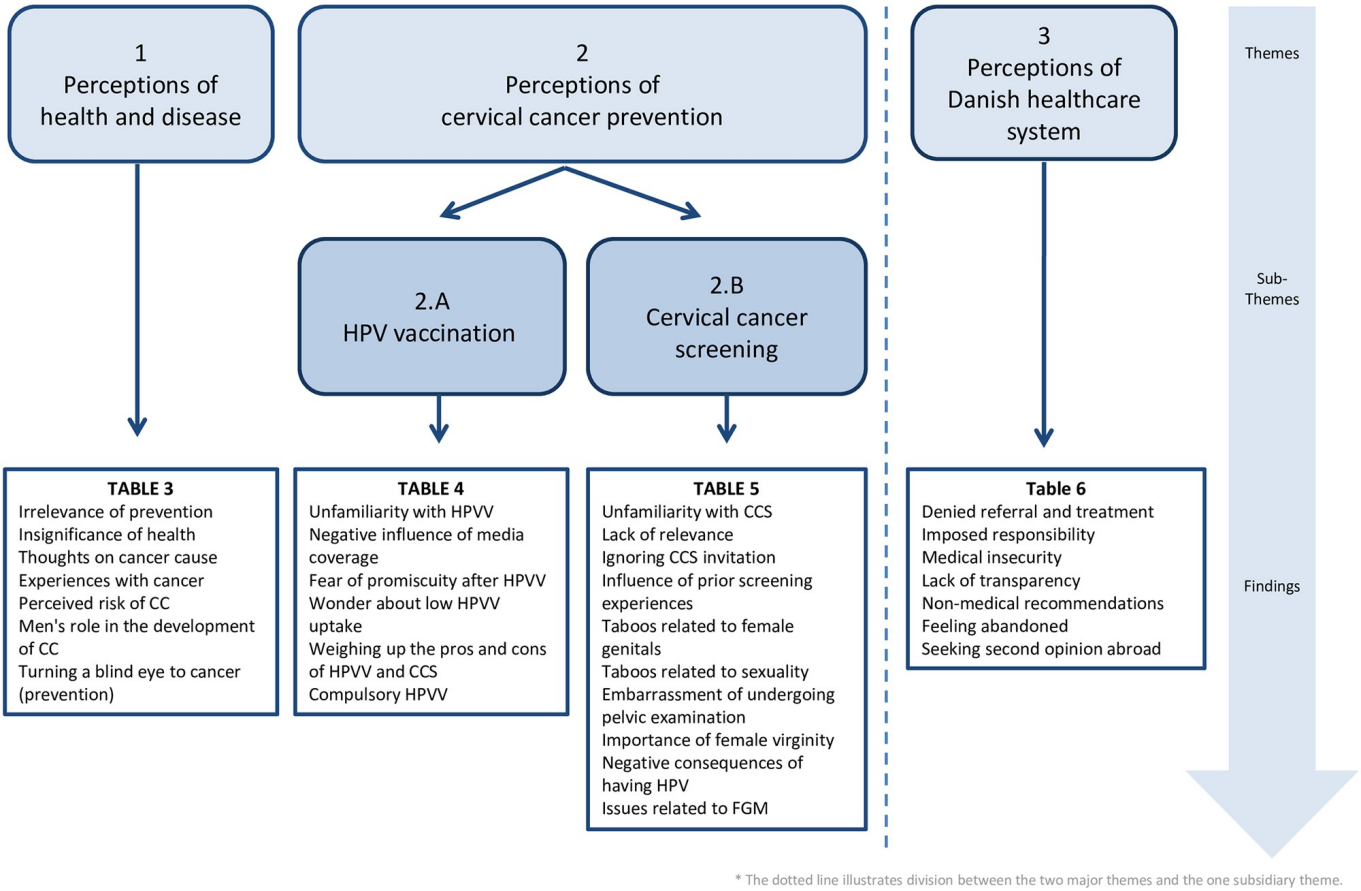
Overview of themes, sub-themes and findings.

Following themes emerged from the STC analysis of the data: (1) ’Perceptions of health and disease; (2) ’Perceptions of CC prevention’ (subdivided into two sub-themes (2.A) ’Perception of HPVV’ and (2.B) ’Perceptions of CCS) and (3) ’Perceptions of the Danish healthcare system’, the latter having a more subsidiary character–as it was not a direct aim of the study—than the preceding two themes. An overview of themes, sub-themes and findings are illustrated in Fig 1.

### (1) Perceptions of health and disease

Insufficient knowledge regarding HPV-induced CC consisted of a lack of awareness concerning aetiology, transmission pathways of HPV, and thus perceived risk factors. Many were convinced that CC was an inherited disease; some believed that cancer aetiology was unknown and that it developed without previous signs/symptoms.

*If you have someone in the family [with cervical cancer]*, *you have a higher tendency to have it*.

*(P9*, **Thoughts on cancer cause**)

-

*Cancer never shows a sign*. *It just comes suddenly*… *just suddenly someone can die*.

*(P3*, **Thoughts on cancer cause**)

When presented to the causal link between HPV infection and cervical cancer, participants focused strongly on the contribution of men in transmitting HPV.

*So (HPV) virus*, *I get it from my husband*, *or?*

*(P7*, **Men’s role in the development of CC***)*

-

*The thing about cell changes—if I was with my husband—and I am 100% sure he’s been a virgin*. *So*, *haven’t I got that cell change?* *If he’s been a virgin*.

*(P9*, **Men’s role in the development of CC***)*

-

*After all*, *they (the men) have it (HPV)… [laughs]* … *why should we have it (the vaccine)?*

*(P16*, **Men’s role in the development of CC***)*

Consequently, most of them felt that they were not susceptible, if they had no family history of CC and were in monogamous and faithful relationships. This low perceived risk resulted in a low relevance of CC prevention.

*There are some people who will think "I have only one partner "*, *so I don’t get it (HPV infection)*.

*(P8*, ***Perceived risk of CC****)*

Furthermore, most friends and relatives they knew with cancer had died from it, making them question the prospects of preventing cancer.

*Those who come from where we come from*, *most of them*, *they go (to the doctor) when it’s already too late*.

*(P2*, **Experiences with cancer***)*

-

*Because everyone who gets the disease*. *They are DEAD*.

*(P7*, **Experiences with cancer***)*

Some questioned the very relevance of disease prevention when not sick.

*If you are not sick*, *why should you check it out?*

(P6, **Irrelevance of prevention**)

Repeatedly, they stated that cancer normally didn’t occupy their everyday lives, only conquering their attention if they experienced the disease themselves or if friends or relatives were diagnosed.

*But I think it is the indifference that one has actually grown up with*. *One does not take one’s health so seriously*. *Especially not in our country*, *you don’t*.

*(P11*, **Insignificance of health***)*

-

*It (cancer) is not something that has really sparked my interest*. *Now*, *we had an aunt who passed away two years away from cancer*, *so we started to get a bit more information on it*.

*(P11*, **Insignificance of health***)*

Even at the end of the FGI´s when the participants’ awareness of CC prevention was improved, one woman stated not wishing to attend CC prevention, as the negative (mental) consequences of knowing were considered to weigh higher than the positive consequences of early cancer detection.

*If I*, *personally*, *had cancer then I would not want to know*. *Because you go down a lot more mentally when you know it*. *So better to live the days you have*.

(P11, **turning the blind eye to cancer (prevention**))

### (2) Perception of cervical cancer prevention

#### (2.A) Perceptions of HPV-vaccination

At the start of the interviews, many expressed that the only kind of vaccination they knew of were traditional childhood vaccinations (e.g., measles, mumps and rubella vaccines).

*All we know are the usual vaccinations*, *the rest we don’t know*.

*(P2*, **Unfamiliarity with HPVV***)*

Others had associated HPVV with the massive negative media attention regarding the safety of HPV vaccines; which had caused a national crisis in HPVV attendance in Denmark few years earlier and resulted in them being afraid or in doubt whether to (continue to) vaccinate their children against HPV.

*What happened in the media made me*, *at least afraid to send my daughter off (to HPVV*)

*(P6*, **Negative influence of media coverage***)*

For these mothers it seemed that the decision to disrupt or continue HPVV was influenced by whether or not they had received a clear recommendation from their GP. Those who had received a clear and reassuring recommendation from their GP were able to cope with the insecurity of the media storm, and did not disrupt HPVV for their daughters. However, those who didn’t receive adequate recommendations from their GP disrupted HPVV–not only for their daughters who were eligible at the time–but also for their younger children.

Another concern was that vaccinating young girls against a sexually transmitted infection could endorse promiscuous behaviour and provoke a backlash among key individuals in the society (e.g., religious leaders). One particular young mother expressed that she prior to the interview would not have vaccinated her daughter but that she would reconsider doing so now, after becoming aware of the vaccine benefits, while admitting that she probably would continue concealing the purpose of the vaccine in order to hinder premarital sex and multiple partners.

*I could never ever have dreamt of getting my daughter vaccinated for this (HPV) before I came here today*… *I don’t want her to be with anyone until she gets married*. *So I didn’t see it as an opportunity for her to be vaccinated at [the age of] 12*. *(…) But now I actually think it will be good (…*.*) But I will not tell her why she is getting it*.

*(P8*, **Fear of promiscuity after HPVV***)*

Simultaneously, most participants were surprised over the fact that several of their fellow countrymen had declined HPVV for their daughters, comparing HPVV to other childhood vaccinations (e.g., MMR).

*It is strange that one doesn’t get the vaccine*, *because it is just a vaccine in the end*. *You get lots of vaccines*.

*(P12*, **Wonder about low vaccination uptake***)*

Finally, at the end of the FGI´s, many expressed a need for more tailored efforts to improve knowledge and HPVV attendance among their countrymen, advocating for community or school-based HPVV and/or education of children and parents. Furthermore, even advocating for mandatory HPVV.

*Can’t you make it (the vaccination) compulsory?* *You can say it’s just like the other vaccines you get as a child*.

*(P16*, **Compulsory HPVV***)*

In line with this, when considering the pros and cons of HPVV and CCS, many stated that they would choose HPVV over CCS, if they were given a choice.

*So*, *if I were faced with the two choices*, *without really knowing what it meant*, *then I would take a vaccine*.

*(P12*, **Weighing up the pros and cons of HPVV and CCS***)*

#### (2.B) Perceptions of cervical cancer screening

At the start of the interviews, most of the participants stated that they didn’t understand the logic behind CCS, thinking that due to this fact many were “lazy” or had other more important things to take care of than CCS.

*I think that because they are lazy*… *many people are actually too busy to go for such tests*, *and especially when it is that they actually do not REALLY know what it is about*

*(P6*, **Lack of relevance***)*

As a result of this lack of understanding, most stated that they at one point in life had thrown away at least one CCS invitation.

*When I was 23 the first time (I received) the invitation (CCS)*. *But I threw it in the trash*. *All the time*… *[giggles]*.

*(P1*, **Ignoring CCS invitation***)*

-

Some participants born abroad stated this lack of understanding was because prevention was not available in their home country, where most people used to seek medical attention only when feeling ill.

*And you are not used to it… going for such tests … for example, screening. So, in our home country… if you are not really SICK. So, it’s not like you’re getting a service*.

*(P6*, **Unfamiliarity with CCS***)*

Some actually found it a bit amusing that some people would seek medical attention if they did not feel ill.

Many perceived CCS to be painful and embarrassing, and felt that this was the main reason for their non-attendance.

*That I should have the doctor take such a test (CCS)*, *for me it is just … embarrassing*. *Even if it is about my health*. *And I also think that’s why I haven’t taken it very seriously*.

*(P15*, **Embarrassment of undergoing pelvic examination***)*

While other participants expressed, despite the fear of pain, that CCS had turned out to be a “quick” and painless examination.

Across nationalities and age groups, issues linked to female sexuality and genitals were commonly considered to be very private matters or even taboo subjects for discussion.

*To be completely honest*, *I think some of these issues are a little taboo in our culture*. *So I don’t think you really talk about it openly*, *to others*.

*(P12*, **Taboos related to female sexuality***)*

-

*If there was a Pakistani woman who had it (cancer in her genitals), then one will not talk so openly about it, in front of others.… I just think it’s because it’s a WOMEN’S AREA*.

*(P12*, **Taboos related to female genitals***)*

Therefore, issues regarding CCS seemed to challenge the participants on a deeper level than those regarding HPVV. As one woman stated “HPVV is just a jab”. Another example illustrating the potential deeply challenging result of attending CCS, two women discussed that having been screened and found to have precursor lesions and thus a sexual transmitted disease (HPV), could lead to serious consequences in marital relationships, as one could be accused of infidelity. Some had heard of marriages being broken as a result of such infidelity accusations,

*So*, *if I find out I have it (HPV) then my husband has a problem… (laughs)*. *Don’t get me wrong*… *it’s because I haven’t had (other partners)*.

*(P9*, **Negative consequences of having HPV***)*

*Response:*

*But he (your husband) may also think that you have had something or other (partners), so conflict can quickly arise. Exactly. So, there is a lot of culture in it*.

*There are sometimes stories about*, *that is*, *a woman who got this disease*. *Then you hear that she is leaving her husband*. *And often*, *it is sometimes the case that it’s in this way that it gets discovered that someone has been unfaithful*.

*(P8*, **Negative consequences of having HPV***)*

Despite the fact that many of the women expressed that men in their societies were often more sexually liberated, they were certain that the woman would automatically be labelled as the “sinner” in these cases. Putting women at risk of being outcasted.

Furthermore, the fact that CCS requires a pelvic examination seemed to be an obstacle in itself, not only because it was perceived as exposing and embarrassing, but also, because it was perceived as jeopardizing virginity and thus not acceptable prior to marriage.

*Such things (gynaecological examination)*, *you don’t do it until you are (married)(…*.*) So before I got married … yeah*, *at that time I was afraid of losing my virginity*.

*(P15*, **Importance of female virginity***)*

For some of the participants, an examination by a male practitioner was considered to be particularly awkward, while for others the gender of the practitioner was irrelevant.

Exclusively among the Somali participants, pelvic examination was considered challenging, due to mental and physical sequels after female genital mutilations (FGM).

*I come from Somalia; you were circumcised*. *I mean*, *I know it is good to go to the doctor and get some tests*. *But the thoughts you have about down there*,… *I mean*, *to be touched down there*, *I don’t want to*.

*(P5*, **Issues related to FGM***)*

At the end of the FGI´s participants did gradually become more positive towards CCS and some unscreened participants even considered attending CCS in the future. Furthermore, when reflecting on their own development during the interviews, many participants were astonished over their own misperceptions prior to the interview. While others continued to have difficulties distinguishing between cancer prevention and cancer treatment.

Consequently, many women expressed a need for more targeted CCS information (e.g., community based or education in one’s native language) and CCS “nudging” (e.g., reminder services or screening under the auspices of existing healthcare services, e.g., midwifes). However, while many stated that, through these educational and nudging efforts, more women would probably attend CCS, they all agreed that CCS would remain challenging for many women like themselves, and that it would not be possible to overcome all presented barriers to CCS. Making broad improvement of CCS attendance among ethnic minority women a complex and difficult task.

### (3) Perceptions of Danish healthcare services

Mistrust in the Danish healthcare system revealed itself through the participants’ interpretations of healthcare professionals’ (HP) intentions, prioritization and medical skills, as well as frustrations with HP´s manners of communication.

First of all, most of the participants perceived themselves as low frequent users of health care services, and they were therefore especially frustrated when addressing their GP and feeling rejected by the system. This frustration was based on experiences of GPs refusing to treat them in accordance with their expectations, or denying them a referral to a more specialized service.

*People they say we are never referred anywhere*. *And we get no treatment*… *Then there are many who do not want to go to the doctor*.

*(P3*, **Denied referral and treatment***)*

While some participants perceived this rejection as systemic issue (e.g., due to a universally stressed healthcare system that meant consequences for all citizens regardless of ethnicity), others perceived that it was because they were immigrants, living in deprived areas or because they had many children. In other word an issue of discrimination.

Some stated that they felt Danish doctors avoided or rejected taking responsibility as they involved the patient in too difficult medical dissensions, making the participants feel insecure.

*The doctor says “It is such and such*, *but it is YOU who has to make the decision*!*" It was BIG*. *Because I don’t know anything about it (HPVV)*… *it’s a burden to get it back (the responsibility)*, *sometimes people think "No*, *now I don’t want to take on that big responsibility"*

*(P6*, **Imposed responsibility***)*

Another factor that made them insecure was when Danish doctors expressed lack of immediate knowledge of the topic of the consultation or sought information on the computer during the consultations. Such behaviour was interpreted by the participants as medical insecurity.

*Yes*, *I actually called my doctor to ask her*. *Because my daughter had just had one (HPVV)*, *and then all that came in the media*. *So*, *I contacted my doctor and asked her*… *“What happens now?” Now I’m very worried*. *And funnily enough*, *the doctor was herself unaware of whether it was true or not (the possibility of HPVV resulting in adverse side effects) …And of course it made me even MORE uncertain*.

*(P6*, **Medical insecurity***)*

Many of the participants also agreed that doctors in Denmark frequently gave “non-medical” recommendations, such as ’drink water’, ’do some sport’, ‘just rest’ or ´wait and see’. These recommendations, seemed inadequate in relation to their expectations and experiences from doctors in their home countries. Therefore, many felt that going to the doctor in Denmark with non-acute issues was a waste of time.

*I only go to the doctor if I am really sick*. *Maybe also because I have experienced going to the doctor*, *I am told that I need to relax and take Panodil (over the counter painkiller—Paracetamol)*. *Do you understand?* *So*, *I feel like maybe I’m wasting my time*.

*(P16*, **Non-medical recommendations***)*

Some had negative experiences from relatives being admitted to Danish hospitals, where they had felt under-prioritized or even abandoned by the hospital staff. As they didn’t feel their relatives were given sufficient attention, treatment and care.

*Also*, *the time when she (my aunt) passed away … we (the family) stood there ourselves with her and she passed away in front of us*… *and it was one and a half or two hours later that a doctor or nurse came to the room …*. *My uncle he simply had to go around the various corridors to find a nurse*. *So*, *for us*, *it seemed like it (my aunt´ situation) was unimportant*. *And they had given up on her*.

*(P11*, **Feeling abandoned***)*

These experiences influenced their trust in the healthcare system to such an extent that they felt reluctant to approach their doctor unless it was absolutely necessary, and resulted in many of the participants seeking second opinion abroad (e.g. either in their home countries and other western countries like Germany and USA).

*She (my doctor in DK) says that I should not take the tablets prescribed in Turkey*. *But I go to a professor in Turkey who was trained in the US who is really good*. *But I shouldn’t take those tablets*, *she says… But she doesn’t treat me (my doctor in DK)*.

*(P3*, **Seeking second opinion abroad***)*

## Discussion

We aimed to explore individual perceptions of and potential barriers to HPVV and CCS among a group of women from MENA countries and Pakistan in Denmark. Initially, we found an overall insufficient knowledge of HPV-induced cervical cancer, and unfamiliarity with CC prevention. When discussing the two preventive measures against cervical cancer, we found that the barriers to HPVV and CCS, respectively, were distinctly different—those related to CCS more often related to socio-cultural barriers. Finally, we observed a general mistrust of the Danish healthcare system that could contribute to hesitancy in attending public health services, such as HPVV and CCS. However, at the end of the interview participants developed a more positive attitude towards CC prevention—especially towards HPVV. Resulting in participants stating that by introducing tailored efforts they believe that many of the presented barriers could be altered. Nonetheless, also stating that not all barriers are adaptable—especially if related to socio-cultural perceptions of female genitals and sexuality.

### Methodological considerations

A qualitative approach making use of FGI constituted a rich, rigorous empirical material, where the generated data proved sufficient to achieve the required information power [23]. Furthermore, thematic analyses (STC) [25] guided the analysis in manageable steps while still ensuring a sufficient analysis to answer the aims of the study.

As we were particularly interested in gaining insight into as many perceptions and barriers as possible, we chose FGI in which we strove to endorse and promote group discussions, in order to elicit as many perspectives as possible and study interactions [21, 22]. To improve representativeness, we included as diverse FGI´s as possible, in some cases we succeeded, while in other cases recruitment turned out to be more difficult than expected and thus some FGI’s were more homogeneous (e.g., FGI3 with four Pakistani relatives). However, in FGI4 we did gain insight into the dynamics between mothers and daughters and between sisters. Which also broadened our perspectives.

We ended up mostly recruiting women who had lived in Denmark for many years, and thus had become accustomed to Danish health care system and traditions, while also possessing knowledge of their cultural background. Therefore, our findings may not cover the presumably greater challenges faced by less acclimatized women (e.g. newly arrived immigrants or socially isolated immigrants). We recruited participants through “snowball” sampling [24], using trustworthy channels such as local ambassadors and bilingual healthcare professionals, which proved effective, and we managed to reach this otherwise hard to reach group of citizens.

Our semi structural interview guide included two time-outs. The argument for including these was to frame the FGI´s and to build a common fundament of knowledge, regarding CC and its prevention, for further discussions. These presentations did expectedly alter the participant’s attitude toward HPVV and CCS. However, they did not modulate all barriers especially not those related to CCS, which indicated that improved knowledge and awareness may have less influence on their CCS behaviour compared to socio-cultural barriers. We were further mindful, in our verbal and non-verbal communication, of the fact that these presentations could introduce a power asymmetry [21] between the researchers and participants.

We were furthermore attentive of our own reflexivity [21] as clinicians and SBE due to her own immigrant background, therefore the data generation, transcriptions and analysis processes were closely monitored by the supervisor LS and the rest of the interdisciplinary authors in the group.

All interviews were conducted, transcribed, and analysed in Danish. Participating in a Danish spoken interview could have made the presentation of complex perceptions and emotional topics difficult for some participants. As a result, the empirical data may lack some nuances, as these could be difficult to express in one’s second language (e.g., the participants use of the phrase “lazy” to explain their nonattendance in CCS). Furthermore, transcribing audio data from bilingual participants may have caused some misinterpretations potentially affecting the analysis. However, including a translator would have introduced other potential challenges which we avoided in the present study. Our findings represent perceptions of women originating from six MENA countries and Pakistan living in Denmark, who have been exposed to the opportunities and traditions of the Danish society, and the results may not reflect immigrants from other regions of the world living in other settings different from the Danish.

The participants sample was contained relatively high proportion of Somali participants, while lacking contribution from some other MENA backgrounds (e.g. Iranian, Kurdish, and Syrian). This could have amplified the challenges faced by Somali women and challenges faced by Iranian, Kurdish, Syrian women could be missing. However, as only the issue of FGM related solely to participants of Somali origin, we do not believe that this had any important implication for the study results.

## Discussion of findings

Many of the presented findings in our study (e.g. insufficient knowledge of CC beside unfamiliarity with and lack of understanding of CC prevention), has also been observed in several other studies conducted among immigrants in different western countries (e.g. USA, Australia, Canada, Europe) [14, 15, 17–19, 26–29]. In many of these studies low language skills also served as an important barrier, a finding we did not encounter due to our purposively selection of participants who spoke fluently Danish. Thus, our findings do not exclude language as a barrier for all ethnic minority women in Denmark, particularly not newly arrived or isolated women.

In our study we further observed HPVV-specific barriers such as; fear of possible adverse vaccine side-effects and fear of promiscuity among daughters after HPVV. Fear of HPVV side-effects was also previously observed among native and immigrant women from different regions of origin [15, 30, 31]. However, in our current study, the fear of adverse events was directly linked to the massive media coverage seen in Demark a few years earlier [32]–a situation also observed in Japan [33]. A finding that has been presented in a previous survey study among native Danish parent [34]. All of which illustrates the influence of the mass media on public attendance at health services and, although not directly related to the aim of this study, this finding emphasizes the importance of sober and correct public health information. Concerns regarding daughters’ sexual activity after HPVV found in this study was also observed in other European and non-European studies [15, 28], suggesting that this concern may influence immigrants’ decisions regarding HPVV across different countries of origin and in different settings.

Furthermore, we found that the CCS-specific barriers were more often related to cultural, traditional or religious norms, than HPVV. It was a general perception among our participants, that women’s sexuality, genital organs, and issues related to these were taboo subjects in their communities. A finding also presented in a qualitative study among native Lebanese women [35], a study among Somali women in USA [36], beside a systematic review in different international settings among majority Chinese, and Hispanic immigrants [28]. The importance of women´s virginity and the fear of spoiling it through pelvic examination (and thus CCS) found in our study was also observed in a previous US study among muslin immigrants women [37], in a Netherlands study among Somali women [15] a Swedish study among immigrant women from different regions [17], beside a systematic US based review [38]. And further supported by a study among native Iranian women, where the importance of an intact hymen (the membrane culturally linked to virginity) was emphasized in the light of Iranian women’s request for hymenoplasty (restoration of the hymen) [39].

Our findings further suggest that MENA and Pakistani women may perceive attending CCS and having precursor lesions as a challenge, because it could cause shame or accusations of infidelity. Shame in relation to having precursor lesions was also observed in a UK study among both immigrant and UK women [16]. Infidelity accusations in MENA and Pakistani societies can further put women’s lives at risk. The most extreme consequence of infidelity accusations or premarital sex is the so-called “honour killing” [40], the act of killing a woman justified by the dishonour that is brought upon the family. The phenomenon is predominately seen in Middle-Eastern countries, but also among immigrants living in Western countries [41], and is estimated to involve as many as 5,000 killings worldwide annually. Something that puts into stark perspective severity of these concerns raised in our study.

Lastly similar to our study, a US study among Somali immigrants, found negative emotions and physical discomfort related to traditional FGM. An issue faced by the majority of Somali women [36]. All of the above suggesting, that many of these CCS-specific barriers, may also be relevant for other non-western immigrants in diverse settings.

However, some of the barriers found in this study may have a more universal character, also affecting western women (both native and immigrants); for example, the observed insufficient knowledge regarding HPV-induced cervical cancer. This has also been reported in studies among native Danish and UK women [31, 42]. Another example is the present finding of a low perceived relevance of CCS; this has likewise been observed among western immigrants in Sweden [43], beside among native women in Norway [44]. Furthermore, the observed perception of pelvic examinations being humiliating and painful, was also observed among native Norwegian participants [44].

If many of the findings among our participants are also seen among native women and immigrant women from western and other non-western, then a question arises: *What might be the reason for the significant lower HPVV and CCS attendance among women with MENA background compared to other women?*

We find it likely that, besides the aforementioned factors, a combination of cultural-specific health and illness perceptions among MENA and Pakistani women [45], unfamiliarity with Danish health care system and mistrust in the health system as well as health care professional [46] could also have played an role in the observed lower HPVV and CCS attendance.

Based on the above, we believe that we have presented findings of a high level of internal validity. Furthermore, we evaluate that our findings are transferable to MENA and Pakistani women in other parts of Denmark, as well as in other countries that have similar sub-populations, and healthcare system/traditions as Denmark. While some of our findings may in addition be transferable to native and immigrant women from other countries, such as insufficient knowledge, pelvic examinations being humiliating and painful.

At the end of the interviews most participants expressed a need for new strategies to improve CC prevention in their community, and suggested implementing various *educational* or *nudging* strategies such as school-based HPVV, to help individuals overcome their barriers. Most participants agreed that they would choose HPVV over CCS if they were given a choice, and believed that others would do the same. However only a few studies have investigated the interplay between HPVV and CCS perceptions and barriers among non-Western immigrants [15, 17, 19], and to our knowledge, no studies have directly reported this preference for HPVV over CCS.

An international systematic review among different populations, comparing environmental, information and behavioural interventions to increase HPVV, found that educational interventions improved HPVV uptake during the active period, but were not sustainable when used alone [47]. They further found that some nudging strategies, such as a reminder intervention and school-based services were particularly effective.

This was highlighted by a recent Danish study examining the effect of reminder letter in the national vaccination program [48], where the authors concluded that reminders were particularly effective in increasing HPVV among immigrants from non-Western countries (OR 2.02 [1, 57–2, 59]), which accentuates the benefits of such nudging interventions to help non-Western parents maintain compliance with vaccination recommendations. Furthermore, supporting the potential impact of implementing school-based HPVV interventions, a Swedish study investigating HPV uptake in different delivery systems (e.g. school-based and outside-school based programs) among Swedish and non-Swedish participants [49]. Here the authors showed a higher overall uptake in school-based programs (79%) compared to two outside-school programs (48% free-of charge outside-school and 37% partly subsidized outside-school). They also showed a relatively lower HPVV uptake among those with non-Swedish mothers in the two outside school programs than the school-based program. Further, having an immigrant background was associated with lower uptake in all three programs, however also most pronounced in the two outside-school programmes (Hazzard Ratio HR_adj = 0.49 [0.48–0.50] partly self-payment, HR_adj = 0.69 [0.66–0.72] for the free-of charge outside school program, compared to the free-of-charge school-based programme (HR adj = 0.82 [0.81–0.83]. Suggesting that not only could free-of-charge school-based HPVV increase the overall uptake of HPV vaccine but also reduce ethnic inequalities in HPVV. This would most probably also be the case in Denmark.

In light of our findings and previous findings, we suggest that implementing tailored strategies to improve HPVV in addition to existing CCS, represents a potent strategy. Besides HPVV seemingly being more acceptable among ethnic minority women/mothers, the currently available nine-valent HPV vaccines protect against 90% of all cervical cancers, as well as HPVV being offered in new gender-neutral programs. All of which could contribute to more equity, larger herd effect and better overall public protection against HPV-induced diseases.

## Conclusion

We found elements of insufficient knowledge and understanding of HPV, and unfamiliarity with CC prevention among a group of long-time resident MENA and Pakistani women in Denmark. Furthermore, we found that socio-cultural barriers, particularly towards CCS, presented a strong hindering for attendance. Additionally, negative experiences and unmet expectations lessen participants trust in the Danish healthcare system. All of which negatively affected their attendance and underlines the need for new tailored CC preventive strategies for this group. Based on our finding we suggest that future studies develop and evaluate complex interventions aiming to improve HPVV and CCS, including strong user-involvement.

## Supporting information

S1 Fig Combined non-attendance. (PDF)

S1 File Background informationa and form of consent in Danish. (DOCX)

S2 File Background informationa and form of consent in English. (DOCX)

S3 File (PDF)

S4 File (PDF)
