# Supplementary material for: Perceptions of cervical cancer prevention among a group of ethnic minority women in Denmark—A qualitative study
Source: PLoS One. 2021 Jun 1;16(6):e0250816. doi: 10.1371/journal.pone.0250816 (PMC8168878; doi:10.1371/journal.pone.0250816)
Supplement: S1 Fig — (PDF) [file pone.0250816.s001.pdf]

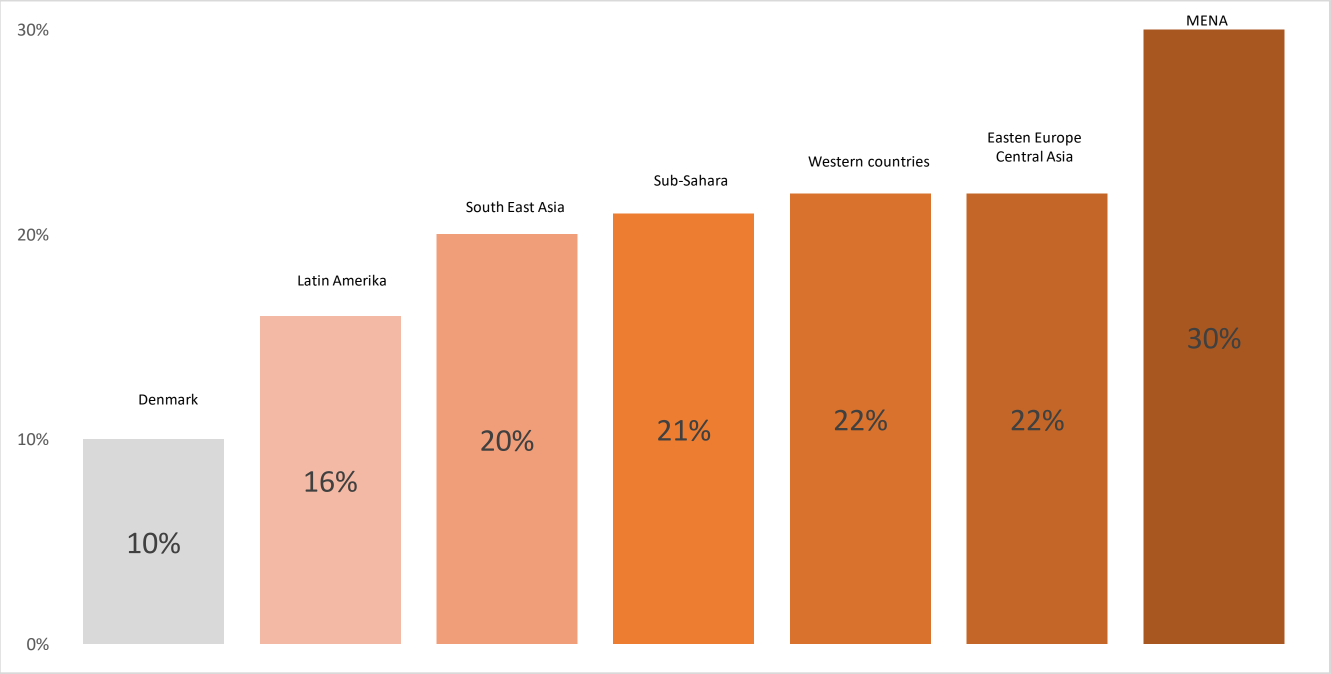

Figure illustrates degree (%) of combined non-attendance among ethnic minority women (by Region of Origin) compared to Danish women ([1])

1. Badre-Esfahani, S., et al., *Low attendance by non-native women to human papillomavirus vaccination and cervical cancer screening - A Danish nationwide register-based cohort study.* Prev Med Rep, 2020. **19**: p. 101106.
