# Supplementary material for: Perceptions of cervical cancer prevention among a group of ethnic minority women in Denmark—A qualitative study
Source: PLoS One. 2021 Jun 1;16(6):e0250816. doi: 10.1371/journal.pone.0250816 (PMC8168878; doi:10.1371/journal.pone.0250816)
Supplement: S1 File — (DOCX) [file pone.0250816.s002.docx]

# -------------------------------------- Baggrundsoplysninger ------------------------------------------

ID: _______

Navn: _____________

Alder: ____

Telefonnummer: _____________

Hvilket land er du født i: _____________

Hvilket land er dine forældre født i: _____________

Hvilket år kom du til Danmark? ________

Hvor i Danmark bor du nu?_____________

Er du gift, samlevende, skilt, enke? (sæt ring om dit svar)

Hvor kommer/kom din mand fra? _____________

Har du børn? Ja Nej (sæt ring om dit svar)

Antal piger:____

Antal drenge: ____

Hvad er din senest afsluttede uddannelse?__________________________________________

Hvad er din mands senest afsluttede uddannelse? ____________________________________

Hvad laver du til daglig? ___________________________________________

Hvad laver din mand til daglig? ___________________________________________

Har du deltaget i screening for livmoderhalskræft? Ja Nej (sæt ring om dit svar)

Har du haft celleforandringer i livmoderhalsen? Ja Nej (sæt ring om dit svar)

Er du HPV vaccineret? Ja Nej (sæt ring om dit svar)

Er jeres døtre vaccineret? Ja Nej (sæt ring om dit svar)

Skal jeres sønner vaccineres? Ja Nej (sæt ring om dit svar)

Hvad mener du er vigtigst?

HPV vaccination eller screening? (sæt ring om dit svar)

**----------Samtykke til deltagelse i sundhedsvidenskabelig undersøgelse-----------**

Jeg har fået både mundtligt og skriftligt information om undersøgelsen.

Jeg ved at det er frivilligt at deltage og at jeg til en hver tid kan trække mit samtykke tilbage.

Jeg giver mit samtykke til at mine oplysninger og udsagn må anvendes i anonymiseret form.

**Deltagers underskrift og dato: _____________________________________________________**

Jeg erklærer at deltager har modtaget skriftligt og mundtligt information om undersøgelsen i tilstrækkelig grad til at deltager kan træffe en beslutning om deltagelse.

**Forskers navn, underskrift og dato:__________________________________________________**
