# Supplementary material for: Perceptions of cervical cancer prevention among a group of ethnic minority women in Denmark—A qualitative study
Source: PLoS One. 2021 Jun 1;16(6):e0250816. doi: 10.1371/journal.pone.0250816 (PMC8168878; doi:10.1371/journal.pone.0250816)
Supplement: S2 File — (DOCX) [file pone.0250816.s003.docx]

# -------------------------------------- Background information ---------------------------------------

ID: _______

Name: _____________

Age: ____

Telephone number: _____________

Which country are you born in? _____________

Which country is your parents born in? _____________

What year did you move to Denmark? ________

Where in Denmark do you live currently? _____________

Are you married, cohabiting, divorced or widower? (circle your answer)

Which country was your husband born in? _____________

Do you have children? Yes No (circle your answer)

How many girls?____

How many boys? ____

What is your latest completed education? __________________________________________

What is your husband´s latest completed education? ____________________________________

What do you do for living? ___________________________________________

What do your husband for living? ___________________________________________

Have your participated in cervical cancer screening? Yes No (circle your answer)

Have your had cell abnormalities? Yes No (circle your answer)

Are you HPV-vaccinated Yes No (circle your answer)

Are your daughters HPV-vaccinated Yes No (circle your answer)

Are your sons HPV-vaccinated Yes No (circle your answer)

What do you think is the most important preventive measure?

HPV vaccination Screening? (circle your answer)

**-------------------Form of consent to participate in scientific project ------------------**

I have received both verbal and written information regarding the project.

I am aware that my participation is voluntary and that I at any time can withdraw my consent to participate

I give consent to the fact that my information and statements may be used in an anonyms form.

**Participants signature and date: _____________________________________________________**

I hereby declare that participants have receives verbal and written information regarding the projects in a sufficient degree in order for the participant to make a decision whether to participate.

**Researchers name and date:__________________________________________________**
