# Supplementary material for: Perceptions of cervical cancer prevention among a group of ethnic minority women in Denmark—A qualitative study
Source: PLoS One. 2021 Jun 1;16(6):e0250816. doi: 10.1371/journal.pone.0250816 (PMC8168878; doi:10.1371/journal.pone.0250816)
Supplement: S3 File — (PDF) [file pone.0250816.s004.pdf]

Udskriftsdato: 28. oktober 2020

LBK nr 1083 af 15/09/2017 (Historisk)

Bekendtgørelse af lov om videnskabsetisk behandling af sundhedsvidenskabelige forskningsprojekter

Ministerium: Sundheds- og Ældreministeriet

Journalnummer: Sundheds- og Ældremin., j.nr. 170622129

Senere ændringer til forskriften

LOV nr 620 af 08/06/2016 § 37 - LOV nr 503 af 23/05/2018 § 22 - LOV nr 726 af 08/06/2018 § 2 - LOV nr 1732 af 27/12/2018 § 2 - LOV nr 1436 af 17/12/2019 § 1 - LBK nr 1338 af 01/09/2020

Lovtidende A

2017 Udgivet den 22. september 2017

15. september 2017. Nr. 1083.

Bekendtgørelse af lov om videnskabsetisk behandling af sundhedsvidenskabelige forskningsprojekter[1)]

Herved bekendtgøres lov nr. 593 af 14. juni 2011 om videnskabsetisk behandling af sundhedsvidenskabelige forskningsprojekter, som ændret ved lov nr. 604 af 18. juni 2012, § 6 i lov nr. 310 af 29. marts 2014, § 37, nr. 1, 6-10, 13-14 og 16, i lov nr. 620 af 8. juni 2016 og § 46 i lov nr. 384 af 26. april 2017.

De ændringer, der følger af § 37, nr. 2-5, 11-12, 15 og 17-43, i lov nr. 620 af 8. juni 2016 om kliniske forsøg med lægemidler, er ikke indarbejdet i denne lovbekendtgørelse, da tidspunktet for ikrafttræden af disse ændringer fastsættes af sundhedsministeren, jf. § 36, stk. 1, i lov nr. 620 af 8. juni 2016 om kliniske forsøg med lægemidler.

Kapitel 1

Lovens formål og område

§ 1. Det videnskabsetiske komitésystem har som formål at sikre, at sundhedsvidenskabelige forskningsprojekter gennemføres videnskabsetisk forsvarligt. Hensynet til forsøgspersoners rettigheder, sikkerhed og velbefindende går forud for videnskabelige og samfundsmæssige interesser i at skabe mulighed for at tilvejebringe ny viden eller undersøge eksisterende viden, som kan berettige forskningsprojektets gennemførelse.

Stk. 2. Det videnskabsetiske komitésystem består af regionale komiteer og en national komité, jf. kapitel 7.

Stk. 3. De videnskabsetiske komiteer er uafhængige og består af medlemmer, der er aktive inden for sundhedsvidenskabelig forskning, og lægmedlemmer, der ikke har en sundhedsvidenskabelig uddannelse og ikke har aktuel tilknytning til sundhedsprofessionerne.

Stk. 4. Loven fastlægger de retlige rammer for komiteernes videnskabsetiske bedømmelse af sundhedsvidenskabelige forskningsprojekter og fastlægger komitésystemets opgaver i forlængelse heraf.

Kapitel 2

Definitioner

§ 2. I denne lov forstås ved:

1) Sundhedsvidenskabeligt forskningsprojekt: Et projekt, der indebærer forsøg på levendefødte menneskelige individer, menneskelige kønsceller, der agtes anvendt til befrugtning, menneskelige befrugtede æg, fosteranlæg og fostre, væv, celler og arvebestanddele fra mennesker, fostre og lign. eller afdøde. Herunder omfattes kliniske forsøg med lægemidler på mennesker, jf. nr. 2, og klinisk afprøvning af medicinsk udstyr, jf. nr. 3.
2) Sundhedsvidenskabeligt forskningsprojekt, der angår kliniske forsøg med lægemidler: Ethvert forsøg på mennesker, der har til formål at afdække eller efterprøve de kliniske, farmakologiske eller andre farmakodynamiske virkninger af et eller flere forsøgslægemidler eller at identificere bivirkninger ved et eller flere forsøgslægemidler eller at undersøge absorption, distribution, metabolisme eller udskillelse af et eller flere forsøgslægemidler med henblik på at vurdere sikkerhed eller effekt af lægemidlet.
3) Sundhedsvidenskabeligt forskningsprojekt, der angår klinisk afprøvning af medicinsk udstyr: Ethvert forsøg på mennesker, der har til formål at afdække eller efterprøve sikkerheden eller ydeevnen af medicinsk udstyr.
4) Multicenterforsøg: Et forsøg, der gennemføres efter en og samme forsøgsprotokol, men på forskellige steder med en forsøgsansvarlig som koordinator eller med forskellige forsøgsansvarlige. Forsøgsstederne kan alle være beliggende i Danmark eller beliggende i Danmark og andre EU-medlemsstater eller tredjelande.
5) Sponsor: En fysisk eller juridisk person, der påtager sig ansvaret for igangsætning, ledelse eller finansiering af et sundhedsvidenskabeligt forskningsprojekt.

1) Loven indeholder bestemmelser, der gennemfører dele af Europa Parlamentets og Rådets direktiv 2001/20/EF af 4. april 2001 om indbyrdes tilnærmelse af medlemsstaternes love og administrative bestemmelser om anvendelse af god klinisk praksis ved gennemførelse af kliniske forsøg med lægemidler til human brug, EF-Tidende 2001, nr. L 121, side 34-44, og dele af Europa Parlamentets og Rådets direktiv 2005/28/EF af 8. april 2005, EU-Tidende, nr. L 91, side 13-19.

CN000450

Sundheds- og Ældremin., j.nr. 170622129

6) Den forsøgsansvarlige: En person, der udøver et erhverv, der er anerkendt til udførelse af forskning, f.eks. via en ansættelse som forsker eller ph.d.-studerende eller på anden vis ved beskæftigelse med konkret forskningsarbejde, og som er ansvarlig for den praktiske gennemførelse af forsøget på et bestemt forsøgssted.
7) Forsøgsprotokol: Et dokument, som beskriver målsætning, udformning, metodologi, tilrettelæggelse, statistiske overvejelser, videnskabsetiske overvejelser, økonomiske forhold, publikationsmæssige forhold, og hvordan deltagere i et sundhedsvidenskabeligt forskningsprojekt vil blive informeret.
8) Forsøgsperson: En person, der deltager i et sundhedsvidenskabeligt forskningsprojekt, uanset om vedkommende er modtager af testpræparater m.v., eller deltager i en kontrolgruppe.
9) Voksen uden handleevne: En person, som er omfattet af definitionen i værgemålslovens § 5, herunder hvor der er iværksat værgemål, der omfatter beføjelsen til at meddele samtykke til deltagelse i sundhedsvidenskabelige forskningsprojekter.
10) Informeret samtykke: En beslutning, der er meddelt skriftligt, dateret og underskrevet eller meddelt elektronisk sammen med brug af digital signatur, om at deltage i et sundhedsvidenskabeligt forskningsprojekt, der efter fyldestgørende information om projektets art, betydning, rækkevidde og risici og modtagelse af passende dokumentation er truffet af egen fri vilje af en person, der er i stand til at give sit samtykke.
11) Stedfortrædende samtykke: En beslutning, der er meddelt skriftligt, dateret og underskrevet eller meddelt elektronisk sammen med brug af digital signatur, om at deltage i et sundhedsvidenskabeligt forskningsprojekt, der efter fyldestgørende information om projektets art, betydning, rækkevidde og risici og modtagelse af passende dokumentation er indhentet fra værgen, forældremyndighedens indehaver eller forsøgsværgen eller fra nærmeste pårørende.
12) Forsøgsværge: En læge, som er uafhængig af den forsøgsansvarliges interesser og af interesser i forsøgsprojekter i øvrigt, kan give stedfortrædende samtykke på forsøgspersonens vegne til deltagelse i et sundhedsvidenskabeligt forskningsprojekt. Forsøgsværgen skal varetage forsøgspersonens interesser.
13) Forskningsbiobank: En struktureret samling af menneskeligt biologisk materiale, der opbevares med henblik på et konkret sundhedsvidenskabeligt forskningsprojekt, og som er tilgængeligt efter bestemte kriterier, og hvor oplysninger, der er bundet i det biologiske materiale, kan henføres til enkeltpersoner.
14) Hændelse: Enhver uønsket hændelse hos en forsøgsperson, der deltager i et forskningsprojekt, efter aktivitet som beskrevet i forsøgsprotokollen, uden at der nødvendigvis er sammenhæng mellem denne aktivitet og den uønskede hændelse.
15) Bivirkning: Enhver skadelig og uønsket reaktion på en aktivitet som resultat af deltagelse i forskningsprojektet.
16) Uventet bivirkning: En bivirkning, hvis karakter eller alvor ikke stemmer overens med den i forsøgsprotokollen beskrevne risiko ved den pågældende aktivitet.
17) Alvorlig hændelse eller alvorlig bivirkning: En hændelse eller bivirkning, som uanset eksempelvis lægemiddeldosis resulterer i død, er livstruende, medfører sygehusindlæggelse eller forlængelse af aktuelt sygehusophold, resulterer i vedvarende eller betydelig invaliditet eller uarbejdsdygtighed eller fører til en medfødt anomali eller misdannelse.

Kapitel 3

Informeret samtykke til deltagelse i sundhedsvidenskabelige forskningsprojekter

§ 3. Sundhedsvidenskabelige forskningsprojekter, der skal anmeldes til komitésystemet efter § 14, kan omfatte forsøg på personer, såfremt forsøgspersonen har givet informeret samtykke hertil forud for deltagelsen i forsøget, jf. dog stk. 2.

Stk. 2. Samtykket skal i stedet gives som et stedfortrædende samtykke efter § 4, hvis forsøgspersonen
1) er mindreårig,
2) er under værgemål, der omfatter beføjelse til at meddele samtykke til deltagelse i sundhedsvidenskabelige forskningsprojekter, jf. værgemålslovens § 5, stk. 1, eller
3) i øvrigt er voksen uden handleevne.

Stk. 3. Samtykket giver sponsor og sponsors repræsentanter og investigator direkte adgang til at indhente oplysninger i patientjournaler m.v., herunder elektroniske journaler, med henblik på at se oplysninger om forsøgspersonens helbredsforhold, som er nødvendige som led i egenkontrol med forskningsprojektet, herunder kvalitetskontrol og monitorering, som disse er forpligtet til at udføre.

Stk. 4. Samtykke til deltagelse i et sundhedsvidenskabeligt forskningsprojekt kan på ethvert tidspunkt tilbagekaldes, uden at det er til skade for forsøgspersonen. En tilbagekaldelse berører ikke adgangen til at behandle personoplysninger, der allerede er indgået i forskningsprojektet, om den pågældende forsøgsperson.

Stk. 5. Ved den videnskabsetiske bedømmelse af konkrete sundhedsvidenskabelige forskningsprojekter kan komiteen fravige stk. 1 og 2 efter de kriterier, der er fastsat i §§ 8-12.

§ 4. Stedfortrædende samtykke på vegne af mindreårige forsøgspersoner skal afgives af forældremyndighedens indehaver.

Stk. 2. Stedfortrædende samtykke på vegne af forsøgspersoner under værgemål, der omfatter beføjelse til at meddele samtykke til deltagelse i sundhedsvidenskabelige forskningsprojekter, jf. værgemålslovens § 5, stk. 1, skal gives af værgen.

Stk. 3. Stedfortrædende samtykke på vegne af øvrige forsøgspersoner uden handleevne skal gives af den nærmeste pårørende og forsøgsværgen.

Stk. 4. Det stedfortrædende samtykke skal være udtryk for forsøgspersonens interesse. Forsøgspersonens tilkendegivelser skal tillægges betydning, i det omfang tilkendegivelserne er aktuelle og relevante.

Stk. 5. Forsøgsværgens stedfortrædende samtykke efter stk. 3 kan ikke påklages til Patientombuddet eller til Sundhedsvæsenets Disciplinærnævn efter lov om klage- og erstatningsadgang inden for sundhedsvæsenet.

§ 5. Samtykke efter §§ 3 og 4 skal være givet på baggrund af skriftlig og mundtlig information om forskningsprojektets indhold, forudselige risici og fordele. Af informationen skal klart fremgå, at samtykket på ethvert tidspunkt kan tilbagekaldes.

Stk. 2. Er forsøgspersonen mindreårig, under personligt værgemål eller i øvrigt voksen uden handleevne, skal informationen tilpasses forsøgspersonens forståelsesevne. Informationen til mindreårige skal meddeles af en person med kendskab til det område, som projektet vedrører, som har de pædagogiske forudsætninger for at formidle informationen til den aldersgruppe, som forskningsprojektet omfatter.

Stk. 3. Informationen nævnt i stk. 1 skal ligeledes meddeles til personer, der er berettiget til at give stedfortrædende samtykke på vegne af en forsøgsperson.

§ 6. Samtykkekravene i §§ 3-5 gælder også for udtagelse af væv og andet biologisk materiale i forbindelse med konkrete forskningsprojekter med henblik på opbevaring i en forskningsbiobank.

§ 7. Sundhedsministeren kan fastsætte nærmere regler om samtykke og information efter §§ 3-5. Medmindre samtykkekrav følger af anden lovgivning, kan sundhedsministeren fastsætte nærmere regler for indhentning af informeret eller stedfortrædende samtykke til brug for anmeldelsespligtige sundhedsvidenskabelige forskningsprojekter, hvori der ikke indgår forsøgspersoner.

Sundhedsvidenskabelige forskningsprojekter, der omfatter afdøde

§ 8. Sundhedsvidenskabelige forskningsprojekter kan indebære brug af væv og andet biologisk materiale fra afdøde udtaget ved obduktion efter sundhedslovens kapitel 56, såfremt der er indhentet samtykke til forskningsmæssig brug af materialet fra den afdøde selv eller fra nærmeste pårørende efter sundhedslovens § 187 om samtykke til lægevidenskabelig obduktion. Væv og andet biologisk materiale udtaget ved retslægelige obduktioner forud for lovens ikrafttræden kan anvendes til sundhedsvidenskabelig forskning uden indhentelse af samtykke efter 1. pkt.

Stk. 2. Sundhedsvidenskabelige forskningsprojekter kan indebære brug af væv og andet biologisk materiale fra afdøde, hvor udtagelsen ikke er omfattet af sundhedslovens kapitel 56 om obduktion, såfremt nærmeste pårørende har samtykket til forskningsmæssig brug af materialet. Nærmeste pårørendes samtykke skal være afgivet på baggrund af skriftlig og mundtlig information om forskningsprojektets indhold.

Dispensation fra samtykkekravet vedrørende 15-17-årige

§ 9. Komiteen kan dispensere fra kravet om stedfortrædende samtykke fra forældremyndighedens indehaver, jf. § 3, stk. 2, nr. 1, og § 4, stk. 1, for en forsøgsperson, der er fyldt 15 år, men ikke er myndig, hvis den pågældende mindreårige selv giver informeret samtykke. Beslutningen om dispensation skal træffes under hensyntagen til forskningsprojektets karakter, risiko og belastning.

Stk. 2. Hvor den mindreåriges eget informerede samtykke vurderes at være tilstrækkeligt, jf. stk. 1, skal forældremyndighedens indehaver have samme information som den mindreårige og inddrages i den mindreåriges stillingtagen.

Registerforskningsprojekter

§ 10. Komiteen kan fravige kravet om samtykke eller stedfortrædende samtykke efter §§ 3-5, hvis et anmeldelsespligtigt sundhedsvidenskabeligt registerforskningsprojekt ikke indebærer sundhedsmæssige risici og forskningsprojektet ikke på anden måde efter omstændighederne i øvrigt kan være til belastning for forsøgspersonen. Det samme gælder, hvis det vil være umuligt eller uforholdsmæssigt vanskeligt at indhente informeret samtykke henholdsvis stedfortrædende samtykke.

Stk. 2. Sundhedsministeren kan fastsætte nærmere regler om de forhold, som er nævnt i stk. 1.

Forskning i akutte situationer

§ 11. Komiteen kan tillade, at et sundhedsvidenskabeligt forskningsprojekt, der ikke angår kliniske forsøg med lægemidler, gennemføres uden forudgående indhentelse af samtykke efter §§ 3-5, hvis forskningsprojektet efter sin karakter kun kan gennemføres i akutte situationer, hvor forsøgspersonen ikke er i stand til at afgive et informeret samtykke, og det ikke er muligt at indhente et stedfortrædende samtykke, hvis

1) deltagelse i forsøget på længere sigt kan forbedre personens helbred eller
2) forsøget kan forbedre tilstanden for andre patienter med samme sygdom som forsøgspersonen og deltagelse i forsøget kun indebærer minimal belastning og risiko for forsøgspersonen.

Stk. 2. Den forsøgsansvarlige skal snarest muligt efterfølgende søge at indhente informeret samtykke eller stedfortrædende samtykke.

§ 12. Komiteen kan tillade, at et sundhedsvidenskabeligt forskningsprojekt, der angår kliniske forsøg med lægemidler, gennemføres uden indhentelse af samtykke efter §§ 3-5, hvis der i stedet indhentes stedfortrædende samtykke fra forsøgsværgen, og hvis forskningsprojektet efter sin karakter kun kan gennemføres i akutte situationer, hvor forsøgspersonen ikke er i stand til at afgive et informeret samtykke, og det ikke er muligt at indhente et stedfortrædende samtykke efter §§ 3 og 4.

Stk. 2. Den forsøgsansvarlige skal snarest muligt efterfølgende søge at indhente informeret samtykke eller stedfortrædende samtykke efter §§ 3 og 4.

Kapitel 4

Anmeldelse af sundhedsvidenskabelige forskningsprojekter

Betydningen af anmeldelse

§ 13. Sundhedsvidenskabelige forskningsprojekter, der er anmeldelsespligtige efter § 14, må ikke påbegyndes uden den kompetente videnskabsetiske komités tilladelse.

Stk. 2. Indebærer et forskningsprojekt kliniske forsøg med lægemidler eller klinisk afprøvning af medicinsk udstyr, der skal godkendes af Lægemiddelstyrelsen, forudsætter komitésystemets tilladelse til gennemførelse af projektet den fornødne tilladelse efter lov om lægemidler henholdsvis lov om medicinsk udstyr.

Anmeldelsespligt

§ 14. Ethvert sundhedsvidenskabeligt forskningsprojekt skal anmeldes til det videnskabsetiske komitésystem, jf. dog stk. 2-5.

Stk. 2. Spørgeskemaundersøgelser og sundhedsvidenskabelige registerforskningsprojekter skal kun anmeldes til det videnskabsetiske komitésystem, såfremt projektet omfatter menneskeligt biologisk materiale.

Stk. 3. Sundhedsvidenskabelige forskningsprojekter, hvori der alene indgår anonymt menneskeligt biologisk materiale, der er indsamlet i overensstemmelse med lovgivningen på indsamlingsstedet, skal kun anmeldes til det videnskabsetiske komitésystem, hvis forskningsprojektet reguleres i § 25 i lov om kunstig befrugtning i forbindelse med lægelig behandling, diagnostik og forskning m.v.

Stk. 4. Forsøg på cellelinjer el.lign., der stammer fra et forsøg med indsamling af celler eller væv, og som har opnået den nødvendige godkendelse, skal kun anmeldes, hvis forsøget angår anvendelse af befrugtede æg, stamceller og stamcellelinjer herfra, jf. § 25 og § 27, stk. 2, i lov om kunstig befrugtning i forbindelse med lægelig behandling, diagnostik og forskning m.v.

Stk. 5. Sundhedsministeren kan fastsætte nærmere regler om de forhold, der i medfør af stk. 4 er undtaget fra anmeldelsespligten. Endvidere kan sundhedsministeren efter indstilling fra den nationale komité fastsætte regler om anmeldelsespligt for nærmere bestemte nye forskningsområder, der ellers ville være undtaget fra anmeldelsespligt i medfør af stk. 4.

Kompetence

§ 15. Anmeldelse af sundhedsvidenskabelige forskningsprojekter skal ske til den regionale komité for det område, hvori den forsøgsansvarlige har sit virke. Sundhedsvidenskabelige forskningsprojekter, der vedrører særlig komplekse områder, skal dog anmeldes til den nationale komité. Anmeldelsespligten påhviler den forsøgsansvarlige og sponsor i forening, og begge skal underskrive anmeldelsen.

Stk. 2. Grænseoverskridende multicenterforsøg skal altid anmeldes her i landet. Er der flere forsøgsansvarlige i Danmark, og er forskningsprojektet ikke omfattet af stk. 1, 2. pkt., skal multicenterforsøget anmeldes til den regionale komité, hvor den koordinerende forsøgsansvarlige har sit virke.

Stk. 3. Sundhedsministeren fastsætter efter indstilling fra den nationale komité nærmere regler om, hvilke sundhedsfaglige forskningsprojekter der i medfør af stk. 1, 2. pkt., skal anmeldes til den nationale komité.

Anmeldelsens udformning

§ 16. Anmeldelse skal ske elektronisk sammen med brug af digital signatur til den kompetente videnskabsetiske komité vedlagt forsøgsprotokol og øvrige oplysninger, der er nødvendige for komiteens vurdering af, om forskningsprojektet kan tillades, jf. § 17, stk. 1, og §§ 18-21, herunder dokumentation for sponsors og den anmeldende forsøgsansvarliges identitet og den forsøgsansvarliges uddannelse.

Stk. 2. Sundhedsministeren fastsætter nærmere regler om krav til udformningen af forsøgsprotokoller, og hvilke øvrige oplysninger der er nødvendige for komitésystemets vurdering, jf. stk. 1, og kan fastsætte nærmere regler om procedurer for anmeldelse.

Kapitel 5

Den videnskabsetiske bedømmelse og komiteernes sagsbehandling

Den videnskabsetiske bedømmelse

§ 17. Komiteerne foretager en videnskabsetisk bedømmelse af indkomne anmeldelsespligtige sundhedsvidenskabelige forskningsprojekter på baggrund af de i loven fastsatte kriterier, herunder §§ 18-22, og en vurdering af, om forskningsprojektet er tilrettelagt på en sådan måde, at der vil blive indhentet og givet fornødent samtykke til forsøgspersoners deltagelse i projektet, jf. kapitel 3. På baggrund af bedømmelsen træffer den kompetente komité afgørelse om, hvorvidt der skal meddeles tilladelse til projektets gennemførelse.

Stk. 2. Den kompetente komité kan fastsætte vilkår for tilladelsen.

Stk. 3. Den kompetente komité fastlægger i forbindelse med en afgørelse om tilladelse, hvordan der skal føres tilsyn med forskningsprojektets gennemførelse, jf. § 28.

§ 18. For meddelelse af tilladelse er det en betingelse, at

1) de risici, der kan være forbundet med at gennemføre projektet, hverken i sig selv eller i forhold til projektets forudselige fordele har et uforsvarligt omfang,
2) den forventede gevinst i terapeutisk henseende og for folkesundheden kan berettige projektet,
3) projektets videnskabelige standard opfylder kravet om, at projektet skal tilvejebringe ny viden eller undersøge eksisterende viden, som kan berettige forskningsprojektets gennemførelse, jf. § 1, stk. 1, 2. pkt., og
4) der er tilstrækkelig grund til at gennemføre projektet, og at forventningerne til projektets konklusioner er berettigede.

Stk. 2. Den kompetente komité skal foretage en afvejning af de forudselige risici og ulemper i forhold til gevinsten for den enkelte forsøgsperson og for andre nuværende og frem-

tidige patienter, herunder om smerter, gener, frygt og anden forudselig risiko minimeres i forhold til forsøgspersonens sygdom og udviklingsstadium, jf. § 1, stk. 1, 2. pkt. Afvejningen skal foretages under hensyntagen til, om forsøgspersonen selv er i stand til at afgive et informeret samtykke, eller om samtykket skal indhentes som et stedfortrædende samtykke.

Stk. 3. Sundhedsministeren kan fastsætte nærmere regler om de forhold, der er nævnt i stk. 1 og 2.

§ 19. For meddelelse af tilladelse til sundhedsvidenskabelige forskningsprojekter, hvori der indgår forsøgspersoner, som er ude af stand til at give et informeret samtykke, er det endvidere en betingelse, at

1) projektet er afgørende for at efterprøve data indhentet ved forsøg på personer, der er i stand til at give et informeret samtykke, eller ved andre forsøgsmetoder,
2) projektet direkte vedrører en klinisk tilstand, som den pågældende befinder sig i, og
3) projektet kan forventes at give patientgruppen en gevinst.

Stk. 2. Er betingelserne i stk. 1 ikke opfyldt, og angår det anmeldte sundhedsvidenskabelige forskningsprojekt ikke kliniske forsøg med lægemidler, er det, jf. dog stk. 3, en betingelse, at

1) projektet ikke med tilsvarende nytte kan gennemføres med inddragelse af forsøgspersoner, der er i stand til at give informeret samtykke, og
2) projektet har udsigt til direkte at gavne forsøgspersonen.

Stk. 3. Er hverken betingelserne i stk. 1 eller 2 opfyldt, og angår det anmeldte sundhedsvidenskabelige forskningsprojekt ikke kliniske forsøg med lægemidler, er det en betingelse, at

1) projektet alene kan gennemføres ved inddragelse af personer omfattet af den pågældende aldersgruppe, sygdom eller tilstand,
2) projektet direkte har udsigt til at kunne medføre meget store fordele for den patientgruppe, som omfattes af samme aldersgruppe, sygdom eller tilstand som forsøgspersonen, og
3) projektet indebærer minimale risici og gener for forsøgspersonen.

Stk. 4. Sundhedsministeren kan fastsætte nærmere regler om de forhold, der er nævnt i stk. 1-3.

§ 20. For meddelelse af tilladelse til sundhedsvidenskabelige forskningsprojekter er det desuden en betingelse, at

1) den forsøgsansvarlige er kvalificeret til at træffe behandlingsmæssige beslutninger og har en behørig uddannelse og klinisk erfaring,
2) det klart fremgår af den skriftlige eller elektroniske information, hvilken økonomisk støtte den forsøgsansvarlige modtager fra private virksomheder, fonde m.v. til gennemførelse af det pågældende forskningsprojekt, og om den forsøgsansvarlige i øvrigt har en økonomisk tilknytning til private virksomheder, fonde m.v., som har interesser i det pågældende forskningsprojekt,
3) eventuelt vederlag eller anden ydelse til forsøgspersonerne for deltagelse i et sundhedsvidenskabeligt forskningsprojekt ikke er egnet til at påvirke samtykkeafgivelsen,
4) forsøgspersonens ret til fysisk og mental integritet samt retten til privatlivets fred respekteres og oplysninger vedrørende forsøgspersonen beskyttes efter lov om behandling af personoplysninger,
5) den forsøgsansvarlige har sikret forsøgspersonen adgang til at få yderligere oplysninger om projektet,
6) projekter, der medfører udførsel af biologisk materiale og oplysninger til tredjelande, gennemføres i overensstemmelse med reglerne i lov om behandling af personoplysninger,
7) der er en erstatnings- eller godtgørelsesordning, hvis forsøgspersonen lider skade eller dør som følge af projektet, og at der er tegnet forsikringer eller foreligger en godtgørelsesordning til dækning af den forsøgsansvarliges og sponsors ansvar over for deltagende forsøgspersoner og
8) der sker offentliggørelse i overensstemmelse med lov om behandling af personoplysninger af såvel negative, inkonklusive som positive forsøgsresultater så hurtigt, som det er muligt og fagligt forsvarligt.

Stk. 2. Sundhedsministeren kan fastsætte nærmere regler om de forhold, der er nævnt i stk. 1.

§ 21. For meddelelse af tilladelse til et sundhedsvidenskabeligt forskningsprojekt, som angår kliniske forsøg med lægemidler, der er omfattet af lov om lægemidler, eller klinisk afprøvning af medicinsk udstyr, der er omfattet af lov om medicinsk udstyr, er det en betingelse, at

1) Lægemiddelstyrelsen er inddraget i godkendelsen af projektet, jf. § 13, stk. 2, og
2) den forsøgsansvarlige er kvalificeret til at træffe behandlingsmæssige beslutninger og har en behørig sundhedsvidenskabelig uddannelse som læge eller, hvor det er relevant, tandlæge og har klinisk erfaring.

Stk. 2. Den kompetente komité skal endvidere påse størrelsen af og de nærmere regler for udbetaling af eventuelt honorar eller eventuel kompensation til den forsøgsansvarlige og forsøgspersoner og indholdet af de relevante klausuler i enhver påtænkt kontrakt mellem sponsoren og forsøgsstedet.

Stk. 3. Sundhedsministeren kan fastsætte nærmere regler om de forhold, der er nævnt i stk. 1.

Konsulentbistand

§ 22. Den kompetente komité skal gøre brug af konsulentbistand i de tilfælde, hvor komiteen ikke selv råder over fornøden faglig ekspertise til at bedømme anmeldte projekter.

Stk. 2. Ved behandling af anmeldte forskningsprojekter, som involverer voksne inhabile, skal forsøgsprotokollen vurderes af en ekspert, der har kendskab til den pågældende persongruppe. Ved behandling af anmeldte forskningsprojekter, som involverer mindreårige, skal forsøgsprotokollen vurderes af en ekspert i pædiatri, hvis den mindreårige indgår i et interventionsforsøg.

Tidsfrister for sagsbehandlingen m.v.

§ 23. Komiteen skal inden for en frist på 60 dage efter modtagelsen af en behørigt udformet anmeldelse, jf. § 16, træffe afgørelse om projektets godkendelse, jf. dog stk. 2-4.

Stk. 2. Fristen i henhold til stk. 1 forlænges med 30 dage, hvis en anmeldelse vedrører forsøg med genterapi, somatisk celleterapi eller lægemidler, der indeholder genmodificerede organismer. Fristen på 90 dage forlænges med yderligere 90 dage i tilfælde af høring af offentlige råd og nævn. Hvis en anmeldelse vedrører forsøg med xenogen celleterapi, gælder der ingen frist for komiteens behandling af anmeldelsen.

Stk. 3. Inden for den periode, hvor anmeldelsen behandles, kan komiteen én gang anmode om oplysninger ud over, hvad ansøgeren allerede har forelagt, med den virkning, at fristen i stk. 1 og 2 afbrydes, indtil de supplerende oplysninger er modtaget.

Stk. 4. Sundhedsministeren kan fastsætte regler, der pålægger de videnskabsetiske komiteer kortere frister for at træffe afgørelse om projekters godkendelse end fastsat i stk. 1-3 mod opkrævning af et særligt gebyr for den fremskyndede sagsbehandling.

Stk. 5. Sundhedsministeren kan efter indstilling fra den nationale komité i særlige tilfælde fastsætte regler om, at behandlingen af forskningsprojekter inden for nærmere bestemte nye forskningsområder, hvori der ikke indgår kliniske forsøg med lægemidler, i særlige tilfælde kan suspenderes for en nærmere angivet periode, indtil en almen etisk eller videnskabelig afklaring har fundet sted.

Beslutningsprocedurer

§ 24. Er det ikke i den kompetente komité muligt at opnå enighed om bedømmelsen af et sundhedsvidenskabeligt forskningsprojekt, træffer komiteen ved afstemning afgørelse om, hvorvidt projektet kan godkendes og der skal meddeles tilladelse til gennemførelse af projektet, jf. dog stk. 3 og 4. Projektet godkendes, hvis et kvalificeret flertal, jf. stk. 2, af medlemmerne i den kompetente komité efter en videnskabsetisk bedømmelse efter § 17 finder, at der kan meddeles tilladelse til forskningsprojektet, eventuelt på vilkår. Ved stemmelighed er formandens eller i tilfælde af formandens forfald næstformandens stemme afgørende.

Stk. 2. Formanden eller i tilfælde af formandens forfald næstformanden skal være en del af flertallet. I en regional komité skal der endvidere være flertal for afgørelsen blandt både lægmedlemmer og sundhedsfaglige medlemmer.

Stk. 3. Fremsætter både lægmænd og sundhedsfagligt udpegede medlemmer og tilsammen mindst en tredjedel af medlemmerne af en regional komité i forbindelse med den regionale komités behandling af en anmeldelse ønske herom, indbringes forskningsprojektet til afgørelse i den nationale komité, der træffer endelig afgørelse.

Stk. 4. Formandskabet for en regional komité kan på den pågældende komités vegne træffe afgørelse i sager, der ikke skønnes at frembyde tvivl.

Meddelelse af tilladelse og orientering herom

§ 25. Den kompetente komité meddeler snarest sin afgørelse om, hvorvidt tilladelse til et sundhedsvidenskabeligt forskningsprojekt kan gives, eller sin afgørelse efter § 24, stk. 3, til den forsøgsansvarlige og sponsor.

Stk. 2. Er afgørelsen truffet af formandskabet, orienteres komiteens øvrige medlemmer om afgørelsen i rimelig tid forud for afgørelsens meddelelse til den forsøgsansvarlige og sponsor.

Stk. 3. Den kompetente komité skal orientere Lægemiddelstyrelsen om afgørelser, der angår kliniske forsøg med lægemidler eller klinisk afprøvning af medicinsk udstyr.

Stk. 4. Den kompetente komité skal orientere øvrige berørte regionale komiteer og den nationale komité om afgørelser om multicenterforsøg.

Klageadgang

§ 26. Den forsøgsansvarlige og sponsor kan i forening senest 30 dage efter modtagelse af afgørelsen indbringe en regional komités afgørelse om tilladelse til at gennemføre et anmeldt forskningsprojekt for den nationale komité til fornyet behandling og afgørelse. Enhver, der i øvrigt er part i sagen, kan senest 30 dage efter afgørelsen indbringe afgørelser fra en regional komité for den nationale komité til fornyet behandling og afgørelse. Klagen indbringes elektronisk sammen med brug af digital signatur.

Stk. 2. Klager over retlige forhold ved afgørelser om forskningsprojekter, der vedrører særlig komplekse områder truffet af den nationale komité, jf. § 15, indbringes for Sundheds- og Ældreministeriet. Stk. 1 finder tilsvarende anvendelse for disse klager.

Behandling af personoplysninger

§ 26 a. En videnskabsetisk komité må kun behandle personoplysninger, som er en del af et sundhedsvidenskabeligt forskningsprojekt, om forsøgspersoner, når behandlingen sker som led i varetagelsen af komiteens tilsyns- og kontrolopgaver efter loven.

Stk. 2. Den, som virker eller har virket i en videnskabsetisk komité eller i et sekretariat for en videnskabsetisk komité, eller som i øvrigt er eller har været beskæftiget med opgaver for komitésystemet, har tavshedspligt om personoplysninger om forsøgspersoner, som den pågældende i den forbindelse har fået kendskab til.

Kapitel 6

Sundhedsvidenskabelige forskningsprojekters gennemførelse, komitésystemets opfølgning og kontrol og øvrige opgaver

Ændringer i godkendte sundhedsvidenskabelige forskningsprojekter

§ 27. Væsentlige ændringer i en forsøgsprotokol for et godkendt sundhedsvidenskabeligt forskningsprojekt må kun iværksættes efter tilladelse fra komitésystemet. Indebærer et forskningsprojekt kliniske forsøg med lægemidler eller klinisk afprøvning af medicinsk udstyr, der skal godkendes af

Lægemiddelstyrelsen, forudsætter komitésystemets tilladelse til iværksættelse af ændringen den fornødne tilladelse efter lov om lægemidler henholdsvis lov om medicinsk udstyr. Sponsor eller den forsøgsansvarlige træffer de fornødne nødsikkerhedsforanstaltninger for at beskytte medvirkende forsøgspersoner.

Stk. 2. Ændringer omfattet af stk. 1 skal anmeldes til den komité, der har meddelt tilladelse til forskningsprojektet. Anmeldelse sker elektronisk sammen med brug af digital signatur vedlagt tillægsprotokol og øvrige oplysninger, der er nødvendige for komiteens vurdering af, om ændringen kan tillades.

Stk. 3. Den kompetente komité træffer efter §§ 17-25 afgørelse om, hvorvidt der kan meddeles tilladelse til ændringen. Dog gælder der en frist for sagsbehandlingen på 35 dage fra modtagelsen af en behørigt udformet anmeldelse.

Stk. 4. § 26 finder tilsvarende anvendelse for afgørelser om ændringer i et godkendt sundhedsvidenskabeligt forskningsprojekt.

Stk. 5. Sundhedsministeren fastsætter nærmere regler om krav til udformningen af tillægsprotokoller og om, hvilke øvrige oplysninger der er nødvendige for komitésystemets vurdering af, om ændringen kan tillades, jf. stk. 2, 2. pkt.

Tilsyn

§ 28. Den regionale komité, der har meddelt tilladelse til gennemførelse af et forskningsprojekt, fører tilsyn med, at et godkendt sundhedsvidenskabeligt forskningsprojekt, der ikke angår kliniske forsøg med lægemidler, udføres i overensstemmelse med denne lov. Er tilladelsen meddelt af den nationale komité, udøves tilsynet af den regionale komité for det område, hvori den forsøgsansvarlige har sit virke, medmindre den pågældende regionale komité på grund af forskningsprojektets kompleksitet anmoder den nationale komité om at varetage tilsynet med et konkret forskningsprojekt. Tilsynet med godkendte sundhedsvidenskabelige forskningsprojekter, der angår kliniske forsøg med lægemidler, udføres af Lægemiddelstyrelsen efter lov om lægemidler.

Stk. 2. Den tilsynsførende komité kan følge et forskningsprojekts forløb og kræve, at den afsluttende forskningsrapport eller publikation sendes til komiteen.

Stk. 3. Den tilsynsførende komité kan som led i tilsynet med et forskningsprojekt, der ikke angår kliniske forsøg med lægemidler omfattet af lov om lægemidler, kræve projektet ændret eller midlertidigt standset eller i særlige tilfælde forbyde forskningsprojektet. Beføjelsen omfatter også anmeldelsespligtige forskningsprojekter, der er iværksat uden fornøden tilladelse.

Stk. 4. Sundhedsministeren kan fastsætte nærmere regler om komiteernes tilsyn og beføjelser efter stk. 1-3.

Tilsynets adgang til oplysninger m.v.

§ 29. Den tilsynsførende komité eller en repræsentant herfor kan påbyde udlevering af alle oplysninger om forskningsprojektet, der er nødvendige for gennemførelsen af tilsynet. Videregivelse og behandling af nødvendige oplysninger om forsøgspersoners helbredsforhold, øvrige rent private forhold og andre fortrolige oplysninger kan som led i tilsynet ske uden forsøgspersonens samtykke.

Stk. 2. Den tilsynsførende komités behandling af personoplysninger som led i tilsynet efter stk. 1 kan kun ske, når det er nødvendigt for at tilgodese hensynet til forsøgspersoners rettigheder, sikkerhed og velbefindende, og når dette hensyn ikke kan tilgodeses ved anvendelse af oplysninger i anonymiseret form. Sundhedsministeren kan fastsætte nærmere regler herom.

Stk. 3. Den tilsynsførende komité eller en repræsentant herfor har til enhver tid mod behørig legitimation og uden retskendelse adgang til virksomheder, sygehuse, praksis og andre steder, der er berørt af forskningsprojektets gennemførelse, for at tilvejebringe oplysninger, der er nødvendige for gennemførelsen af komiteens tilsyn.

Stk. 4. Stk. 3 finder ikke anvendelse på bygninger eller dele af bygninger, der udelukkende anvendes til privat beboelse.

Pligt til underretning om bivirkninger og hændelser

§ 30. Sponsor eller den forsøgsansvarlige skal omgående underrette den tilsynsførende komité, hvis der under gennemførelsen af et sundhedsvidenskabeligt forskningsprojekt opstår formodet alvorlige uventede bivirkninger som følge af projektet. Medmindre forskningsprojektet angår kliniske forsøg med lægemidler omfattet af Lægemiddelstyrelsens tilsyn efter lov om lægemidler, omfatter underretningspligten endvidere alvorlige hændelser.

Stk. 2. Sponsor eller den forsøgsansvarlige skal én gang årligt i hele forsøgsperioden indsende en liste til den tilsynsførende komité over alle formodet alvorlige uventede bivirkninger, som er opstået i forsøgsperioden, og give oplysning om forsøgspersonernes sikkerhed. Medmindre forskningsprojektet angår kliniske forsøg med lægemidler omfattet af Lægemiddelstyrelsens tilsyn i medfør af lov om lægemidler, omfatter underretnings- og oplysningspligten endvidere alvorlige hændelser.

Underretning om afslutning af sundhedsvidenskabelige forskningsprojekter

§ 31. Senest 90 dage efter afslutningen af et anmeldelsespligtigt sundhedsvidenskabeligt forskningsprojekt underretter den forsøgsansvarlige og sponsor i forening den tilsynsførende komité om, at projektet er afsluttet.

Stk. 2. Afbrydes et forskningsprojekt, før det er planlagt afsluttet, underretter den forsøgsansvarlige og sponsor i forening den tilsynsførende komité om afbrydelsen, senest 15 dage efter at beslutningen herom blev truffet. Afbrydelsen skal begrundes. Den tilsynsførende komité kan om fornødent afkræve den forsøgsansvarlige og sponsor en begrundet redegørelse.

Øvrige opgaver

§ 32. Den nationale komité koordinerer arbejdet i de regionale komiteer, fastsætter vejledende retningslinjer og udtaler sig om spørgsmål af principiel karakter, der ikke er knyttet til godkendelsen af et konkret forskningsprojekt.

Stk. 2. Den nationale komité kan foreslå sundhedsministeren at udstede nærmere regler efter bemyndigelserne i denne lov.

§ 33. Komiteerne følger forskningsudviklingen på sundhedsområdet og virker for forståelsen af de etiske problemstillinger, som udviklingen kan medføre i forhold til sundhedsvæsenet og de sundhedsvidenskabelige forskningsmiljøer.

Stk. 2. Komitésystemet skal aktivt samarbejde og koordinere opgavevaretagelsen med relevante nationale og internationale myndigheder og organisationer m.v.

Stk. 3. Komitésystemet skal sikre kvalitetsudvikling, kvalitetssikring og læring i komitésystemet. Sundhedsministeren kan fastsætte nærmere regler herom.

§ 34. De regionale komiteer og den nationale komité afgiver en samlet årsberetning, der indeholder en redegørelse for komiteernes virksomhed og praksis i det forløbne år.

Stk. 2. Årsberetningen, der skal offentliggøres, skal indeholde en liste over alle anmeldte projekter og skal beskrive

1) generelle udviklingstendenser inden for komitésystemets virke, herunder samarbejdet i medfør af § 33, stk. 2, og arbejdet i medfør af § 33, stk. 3, med kvalitetsudvikling, kvalitetssikring og læring i komitésystemet,
2) generelle udviklingstendenser inden for den sundhedsvidenskabelige forskning,
3) væsentlige videnskabsetiske problemer drøftet i komiteerne og
4) begrundelserne for udfaldet af væsentlige sager.

Kapitel 7

Nedsættelse af videnskabsetiske komiteer

De regionale videnskabsetiske komiteer

§ 35. Regionsrådene nedsætter regionale videnskabsetiske komiteer. Et regionsråd kan nedsætte en eller flere komiteer inden for sit geografiske område. En komité kan også nedsættes af flere regionsråd.

Stk. 2. Regionsrådene kan indbyrdes indgå aftale om at fordele indkomne anmeldelser imellem sig.

§ 36. En regional komité består af mindst 7 medlemmer, hvoraf 3 er aktive inden for sundhedsvidenskabelig forskning. Skønner regionsrådet, at hensynet til en regional komités virke, projekternes antal eller andre grunde taler derfor, kan en komité bestå af 9 eller 11 medlemmer. Ved et medlemstal på 9 eller 11 skal 4 henholdsvis 5 medlemmer være aktive inden for sundhedsvidenskabelig forskning.

Stk. 2. Medlemmerne af de regionale komiteer skal have tilknytning til den eller de regioner, den pågældende komité dækker. De forskningsaktive medlemmer udpeges efter indstilling fra relevante forskningsfaglige fora, jf. stk. 7. Indstillingerne skal om muligt indeholde lige mange mænd og kvinder. Ved udpegningen sikres, at der om muligt i komiteen kun er én mere af det ene køn end af det andet.

Stk. 3. Den regionale komité vælger selv sin formand blandt de udpegede forskningsaktive medlemmer og en næstformand blandt de udpegede lægmedlemmer.

Stk. 4. Den regionale komité udarbejder forslag til forretningsorden, der godkendes af National Videnskabsetisk Komité.

Stk. 5. Komiteernes medlemmer udpeges for 4 år ad gangen svarende til den for regionsrådene gældende valgperiode. Genudpegning af medlemmerne kan ske to gange. Der kan udpeges suppleanter for medlemmerne.

Stk. 6. Den afgående komité fortsætter sit virke, indtil nye medlemmer er udpeget og den nye komité er konstitueret.

Stk. 7. Sundhedsministeren kan fastsætte nærmere regler om, hvilke fora der er relevante forskningsfaglige fora, jf. stk. 2.

National Videnskabsetiske Komité

§ 37. Sundhedsministeren nedsætter National Videnskabsetisk Komité.

§ 38. National Videnskabsetisk Komité består af 13 medlemmer, der udpeges på følgende måde:

1) Sundhedsministeren udpeger formanden for National Videnskabsetisk Komité.
2) 2 medlemmer udpeges af sundhedsministeren efter indstilling fra bestyrelsen for Danmarks Frie Forskningsfond.
3) 5 medlemmer udpeges af sundhedsministeren i samråd med ministeren for uddannelse og forskning efter åbent opslag.
4) 5 medlemmer udpeges af sundhedsministeren efter indstilling fra de enkelte regioner.

Stk. 2. Lige mange mænd og kvinder skal om muligt indstilles efter stk. 1, nr. 2, henholdsvis stk. 1, nr. 4. Indstillingen efter stk. 1, nr. 4, skal indeholde lige så mange lægpersoner som personer, der er aktive inden for sundhedsvidenskabelig forskning.

Stk. 3. Ved udpegningen skal det sikres, at komiteen bliver bredt sammensat af medlemmer, der er aktive inden for den sundhedsvidenskabelige forskning, og lægmedlemmer. Endvidere skal det ved udpegningen sikres, at der i komiteen om muligt kun er én mere af det ene køn end af det andet.

Stk. 4. Formanden skal repræsentere statslige forskningsinteresser og folkeoplysende, videnskabsetiske, almenkulturelle eller sociale interesser, der er af betydning for den nationale komités virke.

Stk. 5. Medlemmerne udpeget efter stk. 1, nr. 2, og stk. 1, nr. 3, skal repræsentere forskningsinteresser og folkeoplysende, videnskabsetiske, almenkulturelle eller sociale interesser, der er af betydning for den nationale komités virke.

Stk. 6. Formanden og medlemmerne udpeget efter stk. 1, nr. 2 og 3, må ikke have sæde i Folketinget eller i regionale eller kommunale råd.

Stk. 7. Komiteen vælger selv sin næstformand blandt de udpegede medlemmer.

Stk. 8. Komiteen udarbejder forslag til forretningsorden, der godkendes af sundhedsministeren.

Stk. 9. Komiteens medlemmer udpeges for 4 år ad gangen svarende til den for regionsrådene gældende valgperiode. Genudpegning af medlemmerne kan ske to gange. Der kan udpeges suppleanter for medlemmerne.

Stk. 10. Den afgående komité fortsætter sit virke, indtil nye medlemmer er udpeget og den nye komité er konstitueret.

Kapitel 8

Finansiering

§ 39. Udgifterne til de regionale komiteer afholdes af vedkommende regionsråd.

Stk. 2. Til delvis dækning af udgifterne betaler forskningsinstitutioner m.v. samt private firmaer og hospitaler et gebyr pr. projekt eller ændring, der anmeldes til vedkommende region. Sundhedsministeren fastsætter regler om gebyrets størrelse og opkrævning af gebyret.

Stk. 3. Medlemmerne af de regionale komiteer og deres eventuelle suppleanter modtager udgiftsgodtgørelse efter reglerne i regionslovens § 11.

Stk. 4. Sundhedsministeren fastsætter nærmere regler om regionernes adgang til at yde supplerende udgiftsgodtgørelse til medlemmer af de regionale komiteer og eventuelle suppleanter for disse.

§ 40. Udgifterne til National Videnskabsetisk Komité afholdes af Sundheds- og Ældreministeriet. Sundhedsministeren stiller den fornødne sekretariatsbistand til rådighed for den nationale komité.

Stk. 2. Til delvis dækning af udgifterne til behandling af anmeldelser vedrørende særlig komplekse områder, jf. § 15, stk. 1, betaler forskningsinstitutioner m.v. samt private firmaer og hospitaler et gebyr pr. projekt eller ændring, der anmeldes til National Videnskabsetisk Komité. Sundhedsministeren fastsætter regler om gebyrets størrelse og opkrævning af gebyret.

Stk. 3. Formanden og næstformanden for National Videnskabsetisk Komité og de 7 medlemmer udpeget efter § 38, stk. 1, nr. 2 og 3, og eventuelle suppleanter for disse vederlægges efter aftale med sundhedsministeren, som endvidere afholder udgifterne hertil.

Stk. 4. Medlemmer af National Videnskabsetisk Komité udpeget efter § 38, stk. 1, nr. 4, og eventuelle suppleanter for disse ydes diæter, erstatning for dokumenteret tabt arbejdsfortjeneste og udgiftsgodtgørelse efter reglerne i § 16 a i den kommunale styrelseslov. Udgifterne hertil afholdes af det regionsråd, der har indstillet det pågældende medlem til komiteen.

Kapitel 9

Straf og godtgørelse

§ 41. Medmindre højere straf er forskyldt efter den øvrige lovgivning, straffes med bøde eller fængsel indtil 4 måneder den, der

1) iværksætter et projekt i strid med §§ 13, 14 eller 27 eller iværksætter et projekt i strid med vilkår for tilladelsen, jf. § 17, stk. 2,
2) undlader at efterkomme underretningspligten efter § 30,
3) undlader at efterkomme et påbud eller en oplysningspligt, der har hjemmel i § 28, stk. 3, eller § 29, stk. 1,
4) overtræder forbud, der er nedlagt efter § 28, stk. 3, eller
5) nægter repræsentanter for tilsynsmyndigheden adgang i medfør af § 29, stk. 3.

Stk. 2. I forskrifter, der udstedes i medfør af loven, kan der fastsættes straf i form af bøde for overtrædelse af bestemmelser i forskriften.

Stk. 3. Der kan pålægges selskaber m.v. (juridiske personer) strafansvar efter reglerne i straffelovens 5. kapitel.

§ 42. Sponsor eller, hvis denne ikke har værneting i Danmark, den forsøgsansvarlige, skal betale en godtgørelse på 1.350 kr. til personer, som har deltaget i et projekt iværksat i strid med §§ 13, 14 eller 27, eller som ikke har afgivet informeret samtykke, eller hvor der ikke er indhentet stedfortrædende samtykke, jf. §§ 3-5 og § 12, medmindre den forsøgsansvarlige eller sponsor kan dokumentere, at forholdet ikke skyldes fejl fra den forsøgsansvarliges eller sponsors side.

Stk. 2. Godtgørelsens størrelse reguleres årligt pr. 1. januar med 2,0 pct. tillagt tilpasningsprocenten for det pågældende finansår, jf. lov om en satsreguleringsprocent. De herefter fremkomne beløb afrundes til nærmeste med 50 delelige kronebeløb. Reguleringen sker på grundlag af de på reguleringstidspunktet gældende beløb før afrunding.

Stk. 3. Godtgørelse fastsættes på grundlag af de beløb, der var gældende på det tidspunkt, hvor forsøgspersonen deltog i projektet.

Stk. 4. Bestemmelsen i stk. 1 berører ikke en persons adgang til eller størrelsen af erstatning efter dansk rets almindelige regler.

Kapitel 10

Ikrafttræden, overgangsordning m.v.

§ 43. Loven træder i kraft den 1. januar 2012 og finder anvendelse for sundhedsvidenskabelige forskningsprojekter, der anmeldes efter lovens ikrafttræden.

Stk. 2. Samtidig ophæves lov nr. 402 af 28. maj 2003 om et videnskabsetisk komitésystem og behandling af biomedicinske forskningsprojekter.

Stk. 3. Regler, der er fastsat i medfør af lov om et videnskabsetisk komitésystem og behandling af biomedicinske forskningsprojekter forbliver i kraft, indtil de ophæves af regler fastsat i medfør af denne lov.

Stk. 4. De hidtil gældende regler finder fortsat anvendelse for sundhedsvidenskabelige forskningsprojekter, der er anmeldt før lovens ikrafttræden.

Stk. 5. Medlemmerne af de nuværende regionale komiteer fortsætter deres virke i de regionale komiteer efter denne lov indtil udløbet af den igangværende udpegningsperiode. Udpegninger til en regional komité efter lov om et videnskabsetisk komitésystem og behandling af biomedicinske forskningsprojekter medgår i opgørelsen af, hvorvidt en person fremover kan udpeges som medlem af en regional komité efter denne lovs § 36, stk. 5.

Stk. 6. Den Centrale Videnskabsetiske Komité nedlægges ved lovens ikrafttræden og erstattes af National Videnskabsetisk Komité.

Stk. 7. Udpegning af medlemmer til National Videnskabsetisk Komité finder sted inden lovens ikrafttræden. Den

første udpegningsperiode forkortes, så den udløber med udgangen af den indeværende regionale valgperiode. Den første udpegningsperiode medgår ikke i opgørelsen af, hvorvidt en person kan genudpeges som medlem af National Videnskabsetisk Komité. Udpegninger til Den Centrale Videnskabsetiske Komité efter lov om et videnskabsetisk komitésystem og behandling af biomedicinske forskningsprojekter medgår ved efterfølgende udpegninger efter denne lov i opgørelsen af, hvorvidt en person kan genudpeges som medlem af National Videnskabsetisk Komité.

§§ 44-48. (Udelades)

§ 49. Loven gælder ikke for Færøerne og Grønland, men §§ 1-45, 47 og 48 kan ved kongelig anordning sættes helt eller delvis i kraft for Færøerne med de ændringer, som de færøske forhold tilsiger.

Lov nr. 604 af 18. juni 2012 (Retsvirkninger af tilbagekaldelse af samtykke samt de videnskabsetiske komiteers behandling af personoplysninger)[1)] indeholder følgende ikrafttrædelses- og overgangsbestemmelse:

§ 2

Stk. 1. Loven træder i kraft den 1. juli 2012.

Stk. 2. Loven finder ikke anvendelse for samtykke til deltagelse i sundhedsvidenskabelige forskningsprojekter, der afgives før lovens ikrafttræden. For et sådant samtykke finder de hidtil gældende regler anvendelse.

Lov nr. 310 af 29. marts 2014 (Ændringer som følge af lov om Danmarks Innovationsfond)[2)] indeholder følgende ikrafttrædelses- og overgangsbestemmelse:

§ 12

Stk. 1. Loven træder i kraft den 1. april 2014.

Stk. 2. Lov om Højteknologifonden, jf. lovbekendtgørelse nr. 834 af 13. august 2008, ophæves.

Stk. 3. Danmarks Innovationsfond indtræder ved lovens ikrafttræden i de rettigheder og forpligtelser, som hidtil har tilkommet Det Strategiske Forskningsråd og Højteknologifonden.

Stk. 4. Følgende bekendtgørelser forbliver i kraft, indtil de ændres eller ophæves:

1) Bekendtgørelse nr. 1170 af 8. oktober 2007 om generelle tilskudsforudsætninger og vilkår for at opnå støtte fra Højteknologifonden samt om afgrænsning af små og mellemstore virksomheder.
2) Bekendtgørelse nr. 1065 af 31. august 2007 om klageadgang over Højteknologifondens afgørelser.

Stk. 5. Bemyndigelser udstedt til Det Strategiske Forskningsråds programkomiteer eller til sekretariatet for Det Strategiske Forskningsråd i medfør af lov om forskningsrådgivning m.v. samt bemyndigelser udstedt til Højteknologifondens sekretariat i medfør af lov om Højteknologifonden forbliver i kraft, indtil de ændres i medfør af lov om Danmarks Innovationsfond.

Stk. 6. Medlemmer af Danmarks Forskningspolitiske Råd fortsætter som medlemmer af Danmarks Forsknings- og Innovationspolitiske Råd indtil udløbet af deres nuværende periode med mulighed for genudpegning, hvis genudpegning kunne ske efter de hidtidige regler. Medlemmer af Danmarks Forskningspolitiske Råd, hvis første periode er udløbet ved lovens ikrafttrædelse, kan udpeges som medlemmer af Danmarks Forsknings- og Innovationspolitiske Råd for en periode på 3 år uden mulighed for genudpegning. Medlemmer af Danmarks Forskningspolitiske Råd, herunder formanden, hvis anden periode er udløbet ved lovens ikrafttrædelse, kan ikke udpeges som medlemmer af Danmarks Forsknings- og Innovationspolitiske Råd.

Lov nr. 620 af 8. juni 2016 om kliniske forsøg med lægemidler[3)] indeholder følgende ikrafttrædelses- og overgangsbestemmelse:

§ 36

Stk. 1. Sundheds- og ældreministeren fastsætter tidspunktet for lovens ikrafttræden. Ministeren kan herunder bestemme, at forskellige dele af loven træder i kraft på forskellige tidspunkter, jf. dog stk. 2.

Stk. 2. § 37, nr. 1, 6-10, 13, 14 og 16, § 38, nr. 1 og 2, og § 40 træder i kraft den 1. juli 2016.

Stk. 3. Loven finder i indtil 3 år efter lovens ikrafttræden ikke anvendelse på ansøgninger, som er anmeldt før lovens ikrafttræden, eller på ansøgninger, som i indtil 1 år efter lovens ikrafttræden anmeldes efter de hidtil gældende regler, jf. forordningens artikel 98. For sådanne ansøgninger finder de hidtil gældende regler anvendelse.

Lov nr. 384 af 26. april 2017 om Danmarks Forsknings- og Innovationspolitiske Råd og Danmarks Frie Forskningsfond[4)] indeholder følgende ikrafttrædelses- og overgangsbestemmelse:

§ 36

Stk. 1. Loven træder i kraft den 1. juli 2017.

Stk. 2. Lov om forskningsrådgivning m.v., jf. lovbekendtgørelse nr. 365 af 10. april 2014, ophæves.

Stk. 3. Bekendtgørelse nr. 322 af 30. marts 2014 om bevillingsfunktionen m.v. under Det Frie Forskningsråd forbliver i kraft, indtil den ændres eller ophæves.

Stk. 4. Bemyndigelser udstedt til faglige forskningsråd under Det Frie Forskningsråd eller til Det Frie Forskningsråds sekretariat i medfør af lov om forskningsrådgivning m.v. forbliver i kraft, indtil de ændres eller ophæves.

Stk. 5. Medlemmer af Danmarks Forsknings- og Innovationspolitiske Råd fortsætter som medlemmer indtil udløbet af deres nuværende periode med mulighed for genudpegning, hvis genudpegning kan ske efter de nye regler.

Stk. 6. Medlemmer af bestyrelsen for Det Frie Forskningsråd fortsætter som medlemmer af bestyrelsen for Danmarks Frie Forskningsfond indtil udløbet af deres nuværende periode med mulighed for genudpegning, hvis genudpegning kan ske efter de nye regler.

Stk. 7. De faglige forskningsråd under Det Frie Forskningsråd fortsætter som stående udvalg efter denne lov, indtil bestyrelsen for Danmarks Frie Forskningsfond beslutter andet.

Stk. 8. Medlemmer af faglige forskningsråd under Det Frie Forskningsråd fortsætter som medlemmer af de stående udvalg under Danmarks Frie Forskningsfond med mulighed for genudpegning, hvis genudpegning kan ske efter de nye regler.

Stk. 9. Klager over afgørelser truffet af faglige forskningsråd under Det Frie Forskningsråd, som er indgivet før lovens ikrafttræden, afgøres efter de hidtil gældende regler.

Sundheds- og Ældreministeriet, den 15. september 2017

ELLEN TRANE NØRBY

/ Emilie Norré Sørensen

[1] Lovændringen vedrører § 3, § 26 a, §§ 29-30 og § 41.
[2] Lovændringen vedrører § 38.
[3] Lovændringen vedrører §§ 1-5, § 9, §§ 11- 13, § 15, § 19, § 21, § 23, §§ 26-28, § 30, § 32, § 34, §§ 41-42.
[4] Lovændringen vedrører § 38.
