# Supplementary material for: Perceptions of cervical cancer prevention among a group of ethnic minority women in Denmark—A qualitative study
Source: PLoS One. 2021 Jun 1;16(6):e0250816. doi: 10.1371/journal.pone.0250816 (PMC8168878; doi:10.1371/journal.pone.0250816)
Supplement: S4 File — (PDF) [file pone.0250816.s005.pdf]

Besvar | Slet Uønsket | •••

SV: Att: Shanna Mejland-Munch. Skriftlig forespørgsel vedr. videnskabelig projekt

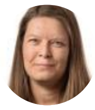

Annette Engsig
ti 28-02-2017, 14:12
Sara Koed Badre-Esfahani

Besvar |

VEK

Markér til opfølgning. Start den 17. juni 2019. Forfalder den 17. juni 2019.

Du videresendte denne meddelelse den 04-05-2020 09:55

Forespørgsel 1/2017

Kære Sara

Du har spurgt De Videnskabsetiske Komitéer for Region Midtjylland om nedenfor beskrevne projekt skal anmeldes til komitéen.

Registerforskning, samt spørgeskemaundersøgelser er undtaget fra anmeldelse i medfør af komitéloven § 14, stk. 2.

Komitéen finder efter en konkret vurdering, at projektet ikke kan betragtes som et sundhedsvidenskabeligt forskningsprojekt, jf. definitionen i komitéloven § 2, nr. 1, og projektet skal derfor ikke anmeldes til komitéen, jf. samme lov § 14, stk. 1.

Du kan derfor gennemføre projektet uden en godkendelse fra komitéen. Du skal være opmærksom på, at du muligvis skal have en godkendelse fra Styrelsen for Patientsikkerhed og/eller Datatilsynet.

Den omtalte lov er lov nr. 593 af 14. juni 2011 om videnskabsetisk behandling af sundhedsvidenskabelige forskningsprojekter.

Med venlig hilsen

Annette Engsig
Sekretær
Juridisk Kontor - De Videnskabsetiske Komitéer for Region Midtjylland

Tel. +45 7841 0185
Annette.Engsig@stab.rm.dk

Regionssekretariatet - Region Midtjylland
Skottenborg 26 - DK-8800 Viborg

Fra: Sara Koed Badre-Esfahani [sarabadr@rm.dk]
Sendt: 04-01-2017 13:43
Til: Komite (Funktionspostkasse) [Komite@rm.dk]
Cc: Marianne Rævsbæk Pedersen [MARAPE@rm.dk]
Emne: Att: Shanna Mejland-Munch. Skriftlig forespørgsel vedr. videnskabelig projekt

Kære Shanna Majland-Munch
Hermed en kort beskrivelse af mit ph.d. projekt. Vil tage stilling til hvorvidt projektet kræver godkendelse fra VEK eller ej.
Mange tak

Vh
Sara Koed Badre-Esfahani

Besvar | Slet Uønsket | •••

[illegible]
den nærmeste fremtid frigives at en ny og mere dækkende vaccine, som beskytter op mod 90 % af tilfælde af livmoderhalskræft. Indtil hele populationen kan blive beskyttet af vaccine, må borgerne være opmærksomme på, at deltagelse i screening for livmoderhalskræft er essentiel for rettidig påvisning og behandling af kræft og -forstadier i livmoderhalsen.
Internationale forskere har udtrykt bekymring for at de HPV-vaccinerede kvinder vil være mindre tilbøjelige til at vælge screeningsundersøgelse efter vaccination. Der er endnu få internationale studier som har undersøgt sammenhængen mellem deltagelse i HPV-vaccination og efterfølgende deltagelse i livmoderhalskræftscreening. Det forestående ph.d. projekt bliver den første nationale danske undersøgelse, der belyser denne sammenhæng.

Nærværende ph.d. studie har som overordnet **formål** at indsamle viden om screeningsdeltagelse hos kvinder i screeningsalderen set i forhold til, om de har til- eller fravalgt HPV-vaccination.

At undersøge associationer mellem valg af HPV-vaccination og efterfølgende deltagelse i screening for livmoderhalskræft hos danske kvinder, der fik tilbud om HPV-vaccination som voksne (*kohorte studie 1*).
At undersøge associationer mellem valg af HPV-vaccination og efterfølgende deltagelse i screening for livmoderhalskræft hos danske kvinder, der fik tilbud om HPV-vaccination som børn (*kohorte studie 2*).

At undersøge motiver for (allerede udført) valg og fravalg af livmoderhalskræftscreening blandt HPV-vaccinerede danske kvinder (*interview studie*).
Kohorte 1. Lukket kohorte studie bestående af alle kvinder født i perioden 1985-1992 og bosat i Danmark i perioden 27. august 2012 til 31. december 2013. *Studieperiode*: 27. August 2012 til 31. December 2013. Eksponeret defineres som modtagelse af minimum en dosis HPV vaccination. Outcome defineres som "deltagelse i screening" ved den første registrerede undersøgelse med prøve af livmoderhalsen. Ved manglende børsteprøve d. 31. december 2016, defineres en borger som "ikke-deltaget". Den detaljerede statistiske analyseplan er under udarbejdelse i samarbejde med biostatistiker ved Aarhus Universitet. Den primære analysemodel bliver en logistisk regression og den sekundære bliver en Poisson regression.
Kohorte 2. Design, eksponering, outcome, metode, datakilder og statistiske analyser er de samme som i korhortestduie 1. *Population*en er alle kvinder født 1993 og bosat i Danmark i 1. januar 2009- 31. december 2015. *Studieperiode*: 1. januar 2009 til 31. december 2019.
Data udtrækkes fra Landspatientregistreret (LPR), Cancerregisteret (CAR), CPR-registret (CPR), Sygesikringsregisteret (SSR), Landsregisteret for Patologi (PAT) samt Lægemiddeldatabasen. Outcome I de to registerbaserede studier er screeningsdeltagelse målt som registreret cytologisk materiale taget fra livmoderhalsen indenfor 1,5 år efter follow-up start.
Interviewstudie. *Design*: Interview studie *Materiale, Datakilde og Metode*: I den fase udvikles viden om HPV-vaccinerede kvinders sundheds- og sygdomsopfattelser, samt personlige overvejelser vedrørende deltagelse i screeningsprogrammet for livmoderhalskræft. Fundene fra fase 3 forventes således både at fungere hypotesegenererende samt forklarende i forhold til resultaterne af de første to kohortestudier. Der anvendes kvalitativ metode i form af henholdsvis fokusgruppe- og individuelle interviews. Den estimerede deltagelsesantal er 10-20 og disse udvælges strategisk via CPR registeret til at repræsentere regionale, kulturelle og socioøkonomiske forskelle i det danske samfund. Potentielle deltagere kontaktes via brev og såfremt der gives tilladelse, kontaktes de telefonisk med henblik på aftale om interview. *Dataanalyse* foretages ud fra en tekstanalytisk metode, hvor temaer udledes og fortolkes kritisk med inddragelse af undersøgelsens teoriramme om folkesundhed og brugerinvolvering(42-44) (47-49).
